# Aspiration-assisted freeform bioprinting of prefabricated tissue spheroids in a yield-stress gel

**DOI:** 10.1038/s42005-020-00449-4

**Published:** 2020-10-16

**Authors:** Bugra Ayan, Nazmiye Celik, Zhifeng Zhang, Kui Zhou, Myoung Hwan Kim, Dishary Banerjee, Yang Wu, Francesco Costanzo, Ibrahim T. Ozbolat

**Affiliations:** 1 Engineering Science and Mechanics Department, Penn State University, 212 Earth-Engineering Sciences Bldg., University Park, PA 16802, USA.; 2 The Huck Institutes of the Life Sciences, Penn State University, 101 Huck Life Sciences Bldg., University Park, PA 16802, USA.; 3 Biomedical Engineering Department, Penn State University, Chemical and Biomedical Engineering Bldg., University Park, PA 16802, USA.; 4 Materials Research Institute, Penn State University, University Park, PA 16802, USA.; 5 Department of Neurosurgery, Penn State College of Medicine, Hershey, PA 17033, USA.; 6 Present address: Department of Cardiothoracic Surgery, Stanford University, Stanford, CA 94035, USA.; 7 Present address: School of Mechatronics Engineering, Nanchang University, Nanchang 330031, China.; 8 Present address: School of Mechanical Engineering and Automation, Harbin Institute of Technology, Shenzhen 518055, China.

## Abstract

Bioprinting of cellular aggregates, such as tissue spheroids, to form three-dimensional (3D) complex-shaped arrangements, has posed a major challenge due to lack of robust, reproducible and practical bioprinting techniques. Here, we demonstrate 3D aspiration-assisted freeform bioprinting of tissue spheroids by precisely positioning them in self-healing yield-stress gels, enabling the self-assembly of spheroids for fabrication of tissues. The presented approach enables the traverse of spheroids directly from the cell media to the gel and freeform positioning of the spheroids on demand. We study the underlying physical mechanism of the approach to elucidate the interactions between the aspirated spheroids and the gel’s yield-stress during the transfer of spheroids from cell media to the gel. We further demonstrate the application of the proposed approach in the realization of various freeform shapes and self-assembly of human mesenchymal stem cell spheroids for the construction of cartilage and bone tissues.

Organ shortage has become more problematic despite an increase in willing donors^1,2^. Three-dimensional (3D) bioprinting has been making a revolutionary impact on life sciences, which has great potential to facilitate fabrication of tissues and organs not only for transplantation but also for drug testing, cancer, or disease modeling^3^. Despite the great progress in bioprinting since early 2000s, the majority of research was devoted to bioprinting in air, limiting the ability to preserve the shape of bioprinted constructs^4^. In this regard, Angelini et al. nurtured the concept of 3D bioprinting within Carbopol microgel acting as a yield-stress support gel in 2015^5^. Around the same time, Feinberg et al. demonstrated the bioprinting within a yield-stress gel consisting of gelatin microparticles, referred to as freeform reversible embedding of suspended hydrogels, which enabled the bioprinting of various different bioinks, such as alginate, collagen, or fibrin^6^. Since then, 3D bioprinting within yield-stress gels or suspension baths exhibiting Herschel-Bulkley or Bingham plastic properties has recently become a powerful approach to create complex-shaped anatomically relevant tissues and organs^5–13^. Due to its shear thinning and self-healing properties, the yield-stress gel transforms from a stable solid state into a flowing fluid phase when exposed to an external stress that exceeds its yield stress^14–18^. As the nozzle moves inside the gel, the gel locally fluidizes when in contact with the nozzle but then rapidly solidifies after the nozzle has passed, eventually acting as a support bath for bioprinted tissue constructs^5,6^. In most cases of bioprinting in a yield-stress gel, cells are bioprinted while being encapsulated within a hydrogel formulation, resulting in limited cell densities compared to the native tissue^19^. Although cell-laden hydrogels can be used to mimic the anatomical configuration easily compared to scaffold-free bioprinting approaches (such as using spheroids^20,21^ or tissue strands^22^ as building blocks), the non-tunable degradation of hydrogels and limited cell-cell interactions (as the cells are confined when encapsulated in hydrogels) are some of the impediments of many such scaffold-based approaches, which was comprehensively evaluated earlier^23^.

Several spheroid bioprinting techniques have been reported in the literature^24–27^. One of the very first and widely explored techniques was extrusion-based bioprinting^24^, in which spheroids were loaded in a syringe barrel and extruded in a delivery gel medium in a controlled fashion. However, spheroids self-assemble readily in the syringe and are prone to break apart during the extrusion process. Concurrently, support structures need to be 3D printed to facilitate the aggregation of extruded spheroids. An important advancement has been made by utilizing the Kenzan method^28^, where spheroids are skewered on a needle array. Since the position of each spheroid depends on the needle size, location, and arrangement, freeform (i.e., complex shaped) bioprinting of spheroids is quite challenging as the spheroid positioning of spheroids along the *z*-axis (direction parallel to the needles) is not independent in each layer. Drop-on-demand bioprinting has also been reported to deposit spheroids^29^. In this approach, spheroids are encapsulated within gel droplets. As such, drop-on-demand bioprinting has inherent limitations on the precision of the 3D bioprinting process. To overcome some of major the challenges of current techniques, we recently demonstrated an aspiration-assisted bioprinting (AAB) technique^20,21^ enabling precise bioprinting of spheroids into or onto sacrificial or functional gel substrates. However, freeform bioprinting of spheroids in 3D has been a long-standing problem due to the layer-by-layer-building nature of the existing techniques.

In this work, we combine cutting-edge advances in AAB of spheroids^20^ and bioprinting within yield-stress gels^5,6^ to enable a new direction in scaffold-free future bioprinting effort, and demonstrate the freeform bioprinting of tissue spheroids by precisely positioning them in self-healing biologically inert yield-stress gels in 3D allowing for the subsequent self-assembly of the bioprinted spheroids for fabrication of tissues. We have already demonstrated the potential of AAB technique to aspirate and pick spheroids^20^ and, now, taking advantage of the Herschel-Bulkey properties of the yield-stress gels receiving the spheroids, we succeeded in the direct transfer of spheroids from the cell media and their freeform positioning within the yield-stress gels on demand. In order to better understand the response of biologics to the bioprinting process, we studied the underlying mechanism explaining interactions between the spheroids and two different yield-stress gels, including Carbopol and alginate microparticles, during bioprinting. We then explored the potential of our Aspiration-assisted Freeform Bioprinting (AAfB) technique in building complex-shaped configurations and demonstrated multiple applications, including the fabrication of cartilage and bone tissues, throughout this study.

## Results

### Working mechanism of AAfB.

**Fig. 1:**
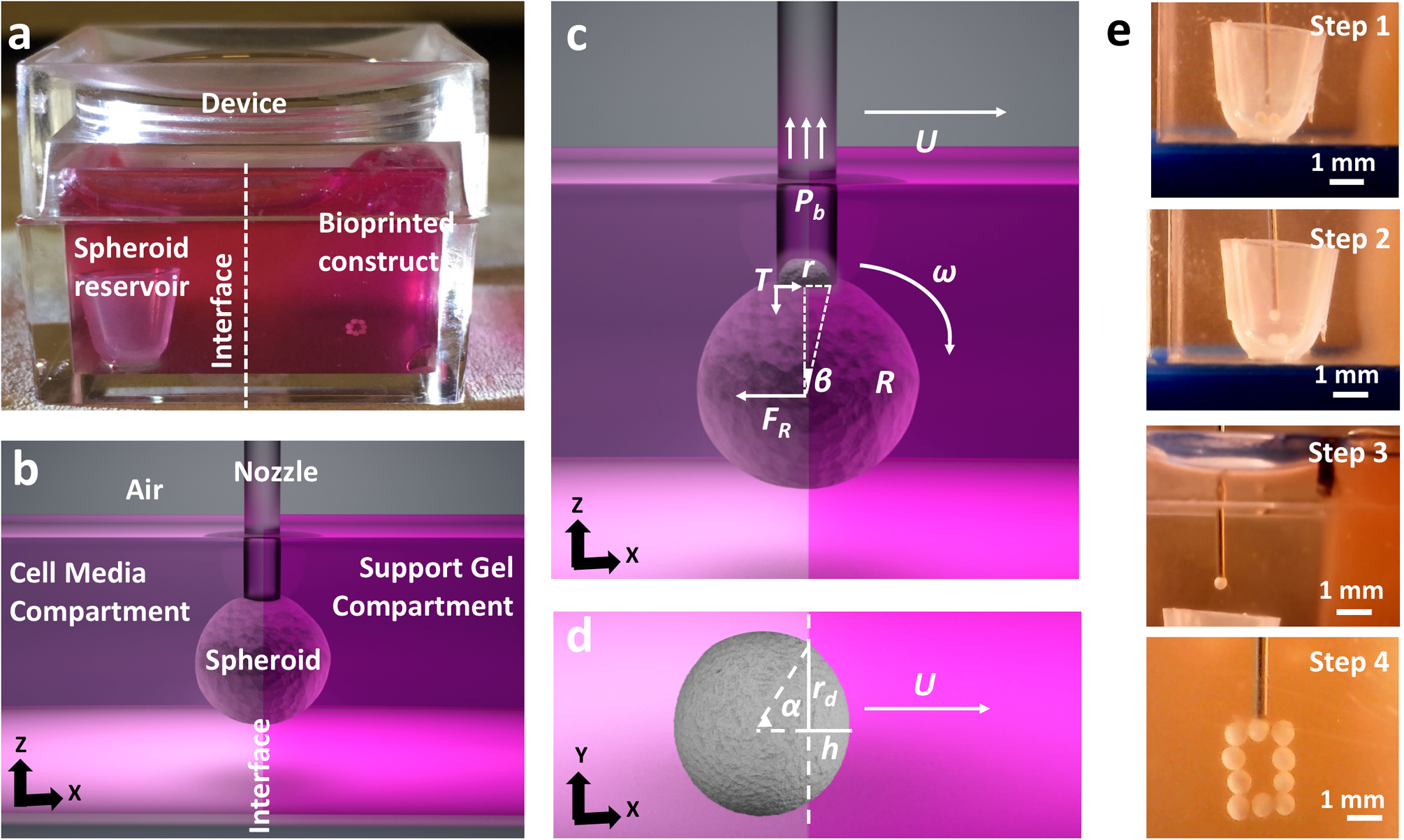
Aspiration-assisted freeform bioprinting process. **a** The bioprinting setup, where a box was filled with the yield-stress gel in one compartment and cell media in the other. **b** A schematic showing the process of spheroid traverse across the yield-stress gel and media compartment. **c**, **d** Schematics showing physical parameters involved in transferring of spheroids from the cell media to the yield-stress gel, *F*_*R*_ is the magnitude of the resultant force acting on the spheroid due to its interaction with the environment, *r* is the nozzle’s radius, *U* is the bioprinting speed, and *P*_*b*_, the critical aspiration pressure, is a function of *R*, *r*, *U*, gel properties (*K*, *n*, *τ*_0_). **e** Images showing a step-by-step illustration of the process, where (Step 1) spheroids were stored in the reservoir in the cell media, (Step 2) spheroids were picked from the reservoir, (Step 3) traversed the interface into yield-stress gel from the cell media compartment and (Step 4) bioprinted to form a predefined shape.

In this study, we further advanced our recently published AAB technique^20,21^ to demonstrate the freeform bioprinting of spheroids within a yield-stress gel. Specifically, aspiration forces were used to pick up spheroids from the spheroid reservoir (placed inside the cell media compartment) and transfer them into the yield-stress gel (occupying inside the yield-stress gel compartment) one by one (Fig. 1a–d). The spheroids were transferred from the cell media through a highly mobile transition interface into the self-healing yield-stress gel. In general, gels have small elasticity and high viscosity, and their mechanical response is usually described by a viscoelastic model^30^. Here, we present some elementary moment balance arguments leading to the estimate of the minimum aspiration pressure that is needed for a spheroid to be transferred from the media to the gel compartment. With reference to Fig. 1e, whether the spheroid was moving through the interface or through the gel, we observed that the spheroid was acted upon by forces due to its interaction with its environment and with the nozzle. If the aspiration pressure fell below a critical value *P*_*b*_, the spheroid would separate from the nozzle, typically by pivoting against the trailing edge of the nozzle (trailing relative to the direction of motion). We label the pivot point by *T* in Fig. 1c. We denote by *F*_*R*_ the magnitude of the resultant force acting on the spheroid due to its interaction with the environment. Referring to Fig. 1c, we observed that at the critical pivot condition, the only forces contributing to the moment about the point *T* are the resultant of the applied aspiration pressure distribution and the force with magnitude *F*_*R*_. With this in mind, we can then estimate the critical aspiration pressure *P*_*b*_ by considering the balance of moments about *T*. Clearly, to proceed to such an estimate, we need to know both the values of *F*_*R*_ and its direction as well as the state of motion of the spheroid. Since *F*_*R*_ represents the resistance offered by the gel to the spheroid’s motion, we make the simplifying assumption that, when the spheroid is moving at a constant speed along a horizontal line, the resistance is also horizontal and with a line of action going through the spheroid’s center. Under these simplified conditions, the moment balance about *T*, ∑*M*_*T*_ = 0, yields the following relation:

(1) $$P_{b}\left(\pi r^{2}\right)r-F_{R}(R\text{cos}\theta )=0,$$

where, with reference to Fig. 1c, d, *r* is the nozzle’s radius and *θ* is such that tan *θ* = *r*/*R*. Solving Eq. (1) for *P*_*b*_, we obtain:

(2) $$P_{b}=\frac{\sqrt{R^{2}-r^{2}}}{\pi r^{3}}F_{R}.$$

Next, we need to provide an estimate for the value of *F*_*R*_. This estimate can be complex in that *F*_*R*_ is determined by different physics depending on the position of the spheroid relative to the interface between the medium and gel compartments. When the spheroid is moving through the gel at a constant speed, it is reasonable to assume that *F*_*R*_ = *F*_*D*_, where *F*_*D*_ is the drag acting on a sphere moving at a constant speed in a viscous fluid under laminar conditions. In fact, treating the spheroid as a rigid particle with a radius *R* (<450 μm), the relevant Reynolds number^31^ is Re = 2*ρ*_gel_*UR*/*η*_0_, where *ρ*_gel_ is the mass density of the gel, which is assumed to be the same as water (as a matter of fact, the mass density of the spheroids can also be assumed to be that of water: *ρ*_*s*_ = *ρ*_gel_ = *ρ*_*w*_), and *U* ~ 2.5 mm s^−1^ is the bioprinting speed (also the speed of the spheroid’s center-of-mass, and *η*_0_ is the gel’s Newtonian equivalent viscosity or zero-shear rate viscosity, measured at 44 and 13.8 Pa s for 1.2% Carbopol and 0.5% alginate microparticles, respectively). Under these assumptions, Re is on the order of ~10^−6^ confirming that the flow around the spheroid during bioprinting is indeed laminar. Under these conditions, we can use the well-known formula *F*_*D*_ = 6*πRUη*_*U*_, where the value of viscosity *η*_*U*_ depends on *U* as the gel is shear thinning^32^.

More complex is the estimation of *F*_*R*_ when the spheroid is traversing the interface between the medium compartment and the gel. In this case, we can distinguish four contributions to *F*_*R*_: again the drag exerted on the spheroid by its surroundings (*F*_*D*_), the resistance provided by the elasticity of the gel below the yield limit as the spheroid is indenting the gel (*F*_*E*_), the thermodynamic force (*F*_*I*_) representing capillary effect at the interface, and nonlinear and dynamic terms (*F*_*N–D*_), neglecting fluctuations and the rotational effects^33^, as the motion cannot be treated as being steady:

(3) $$F_{R}=F_{D}+F_{E}+F_{I}+F_{N-D}.$$

**Fig. 2:**
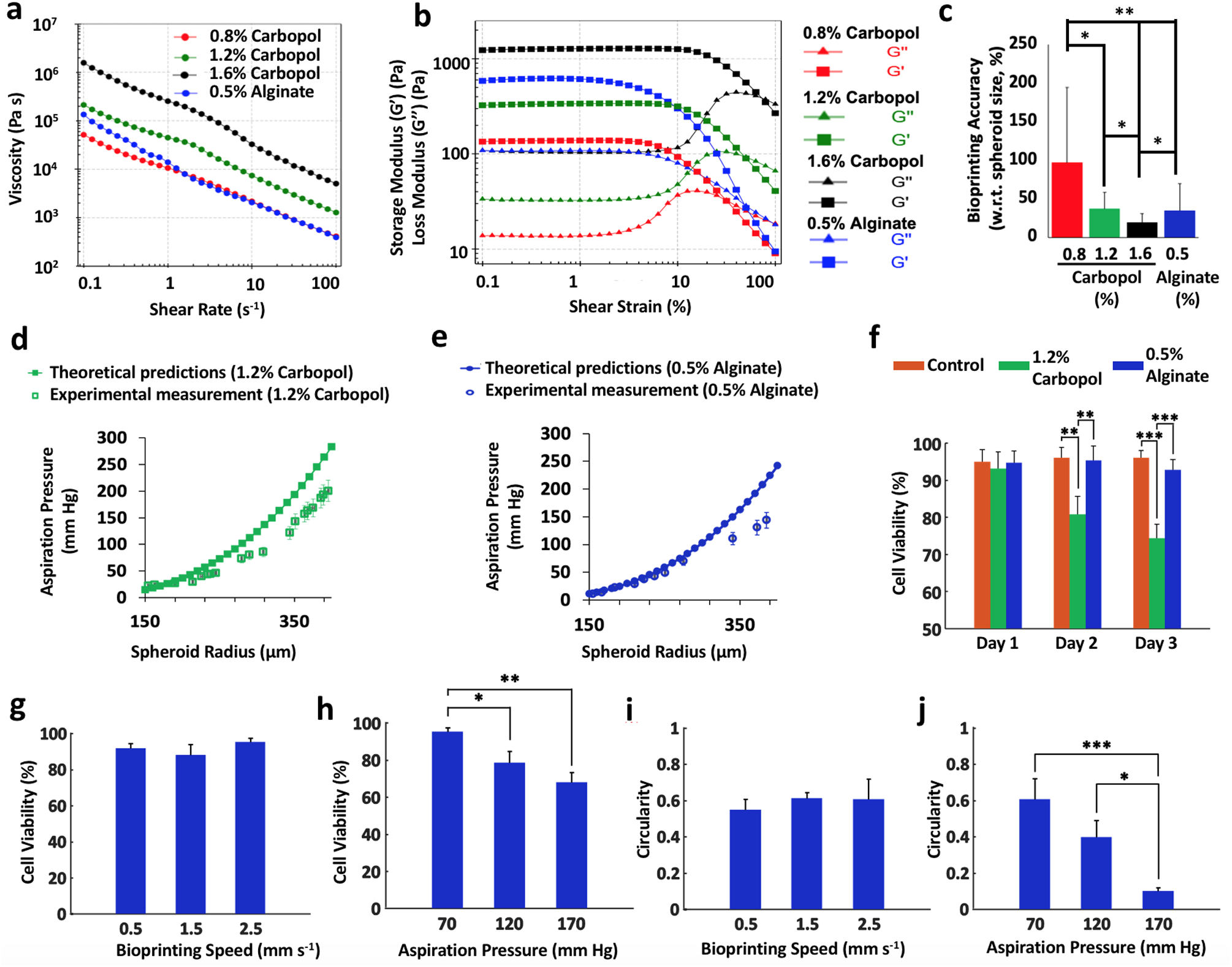
The interplay among gel, spheroid, and bioprinting process parameters and its role in spheroid viability and shape. **a**, **b** Rheological properties of Carbopol at different concentrations and 0.5% alginate microparticles. The gels showed shear-thinning properties indicated by decreasing viscosity with shear rate. **c** The bioprinting accuracy of the yield-stress gels (with respect to spheroid size) (*n* = 5; *p < 0.05 and **p < 0.01). The positional precision for 0.8%, 1.2%, and 1.6% concentrations of Carbopol and 0.5% alginate microparticles were observed to be ~97%, 22%, 12%, and 34%, respectively. The colors correspond to the legend of panel (**a**). **d**, **e** Confirmation of the theoretical approach using the experimental validation for spheroids ranging from 150 to 450 μm in radius bioprinted in 1.2% Carbopol and 0.5% alginate microparticles. Note that human mesenchymal stem cell (MSC) spheroids were utilized in all experiments. The theoretical relation was plotted according to Eq. (6). **f** Cell viability of MSC spheroids in different yield-stress gels over 3 days (note that free standing MSC spheroids were used as a positive control, *n* = 3; **p < 0.01 and ***p < 0.001). **g**–**j** Cell viability and circularity of MSC spheroids at different bioprinting speed and aspiration pressure in alginate microparticles (*n* = 3; *p < 0.05, **p < 0.01 and ***p < 0.001). Increasing the bioprinting speed from 0.5 to 2.5 mm s^−1^ did not reduce the cell viability when the aspiration pressure was maintained constant. However, increasing the aspiration pressure from 70 to 170 mm Hg decreased the cell viability. On the other hand, increase in the bioprinting speed did not significantly change the circularity of spheroids under a given aspiration pressure, whereas, increasing the aspiration pressure from 70 to 170 mm Hg increased the deformation of the spheroids and reduced their circularity. Error bars were plotted as mean ± standard deviation.

Whether in the gel or at the interphase, for simplicity, we will estimate *F*_*D*_ using the same drag formula mentioned earlier scaled to account for the fact that the spheroid is not completely in the gel (Fig. 1d): *F*_*D*_ = *Ur*_*d*_*η*_*U*_ (6*α* + 8sin *α* + sin2 *α*), where *r*_*d*_ is the contact radius and where the advancing angle *α* is defined via the relation tan *α* = *r*_*d*_/(*r*_*d*_ − *h*), *h* being the indentation depth^32^ (Fig. 1d). We feel that this estimate is acceptable in an effort to understand what physics dominates the value of *F*_*R*_. Clearly, the maximum resistance provided by the gel to the spheroid after traversing the interface is *F*_*D*_ = 6*πr*_*d*_*Uη*_*U*_, as shown before. Referring to Fig. 2a, b, our experimental rheological study indicates that the gel should be modeled as a (shear thinning) Herschel-Bulkey fluid with viscosity:

(4) $$\eta_{U}=\frac{\tau_{0}}{\dot{\gamma }}+K{\dot{\gamma }}^{n-1},$$

where *τ*_0_ is the yield stress, $\dot{\gamma }$ is the shear rate which we estimate as $\dot{\gamma }=U/R$ for the motion in the gel or as $\dot{\gamma }=U/r_{d}$ for the motion through the interface, *K* is the consistency index, and *n* is the power-law exponent (*n* < 1 for shear-thinning fluids^34^). Two fluid property constants can be identified from the power-law shear-thinning regime in Fig. 2a, *n* can be obtained by adding one to the slope of the viscosity versus shear rate curve and the consistency index *K* is equal to the viscosity of the gel when the shear rate is equal to 1. From our experiments, we determined that *K* = 44 (Pa s^*n*^) and *n* = 0.3 for 1.2% Carbopol, whereas, *K* = 13.8 (Pa s^*n*^) and *n* = 0.18 for 0.5% alginate microparticles. Thus, $\eta_{U}=44{\dot{\gamma }}^{-0.7}+\tau_{0}/\dot{\gamma }$ and $\eta_{U}=13.8{\dot{\gamma }}^{-0.82}+\tau_{0}/\dot{\gamma }$ are for Carbopol and alginate microparticles, respectively, with a unit of Pa s.

At the initial stage of contact^35^, *F*_*E*_ = 4*πEhR* or using the same geometric configuration as above, *F*_*E*_ = 4π*ER*^2^(1 − cos*α*), *E* being the gel’s Young’s modulus. *E* can be estimated from rheological measurements of the storage shear modulus G′. Specifically, we have *E* ≈ 2*G*′(1 + *v*) = 3G′, where *v* is the Poisson ratio, which, for an incompressible material like Carbopol or alginate microparticles, can be taken to be equal to 0.5^36^. Our measurements of G′ were reported in Fig. 2b. The term *F*_*E*_ is only considered while the spheroid is traversing the interface and neglected when the spheroid is fully submerged in the gel.

Another term is the thermodynamic interfacial force is experienced when the spheroid is traversing the media-gel interface. The maximum value can be estimated to be:

(5) $$F_{I}=2R\sigma_{1,2}{\text{cos}}^{2}\frac{\theta }{2},$$

Here *σ*_1,2_ is the surface tension coefficient between the media and gel. The last term, *F*_*N–D*_, includes the nonlinear and dynamic terms, such as rotation and inertia. Typical values for the surface tension coefficient are a few tens of mN m^−1^. For the $F_{I}/F_{D}=\frac{2R\sigma_{1,2}{\text{cos}}^{2}\frac{\theta }{2}}{6\pi RU\eta_{U}}=\frac{\sigma_{1,2}}{3\pi U\eta_{U}}\sim 0.1$. term, we assume an ideal value of 1 to maximize the interfacial effect. Considering the ratio of interfacial and drag term:

$$F_{I}/F_{D}=\frac{2R\sigma_{1,2}{\text{cos}}^{2}\frac{\theta }{2}}{6\pi RU\eta_{U}}=\frac{\sigma_{1,2}}{3\pi U\eta_{U}}\sim 0.1.$$

Based on the above force analysis, the main contribution to the term *F*_*R*_ is the drag experienced by the spheroid as it moves through the gel, so that:

(6) $$P_{b}\approx \frac{\sqrt{R^{2}-r^{2}}}{\pi r^{3}}6\pi RU\left(\frac{\tau_{0}}{\dot{\gamma }}+K{\dot{\gamma }}^{n-1}\right),$$

*η*_*U*_ is the viscosity at a bioprinting speed of 2.5 mm s^−1^. As a result, *P*_*b*_ is a function of *R*, *r*, *U* gel properties (*K*, *n*, *τ*_0_). We do not include the terms that are a weak function of *E*, *σ*_1,2_ and *θ*, which are negligible compared to the viscosity of the gel. However, while crossing the interface, *F*_*E*_ term should also be included in the estimation of *P*_*b*_.

In order to determine an appropriate gel concentration for AAfB, we preferred to test 0.8, 1.2, and 1.6% concentrations of Carbopol and 0.5% alginate microparticles (with 90% of the particles were observed to have a particle size <89.5 μm (Supplementary Fig. 1)), where such ranges were corroborated with respect to concentration used in previous studies^10,19^. Our rheological experiments demonstrated a yield stress value of 5.3, 25.7, 136.1, and 21.9 Pa for 0.8%, 1.2%, and 1.6% Carbopol and 0.5% alginate microparticles, respectively (Supplementary Fig. 2). All concentrations showed shear-thinning properties indicated by decreasing viscosity with shear rate (Fig. 2a), and solid to fluid transition occurred at ~31%, 48%, 72%, and 47% strain for 0.8%,1.2%, and 1.6% Carbopol, and 0.5% alginate, respectively. Supplementary Fig. 2b shows the frequency dependency of G′ and G″ for all gels, where G′ was more than G″ at the frequency range of 0.1–100 rad s^−1^. In particular, G″ for 0.8 and 1.2% Carbopol gels was observed to be more dependent to frequency at higher frequencies than lower ones indicating that these gels exhibited stronger viscous character at higher frequencies. Figure 2c shows bioprinting positional accuracy with respect to the spheroid size, which was improved with increasing Carbopol concentration such that 1.6%, 1.2%, and 0.8% Carbopol yielded 19%, 37%, and 97% positional accuracy, respectively, whereas 0.5% alginate microparticles showed a positional accuracy of 35%. As shown by the error bars in Fig. 2c, the positional precision for 0.8%, 1.2%, and 1.6% concentrations of Carbopol and 0.5% alginate microparticles were determined to be ~97%, 22%, 12%, and 34%, respectively. Thus, we preferred to use 1.2% Carbopol and 0.5% alginate for further experiments. In order to validate the theoretical approach, we performed bioprinting experiments to establish a relationship between *r* and *P*_*b*_. As indicated in Fig. 2d, e, the theoretical approach was confirmed by the experimental approach and the results were close to each other for both Carbopol and alginate microparticles, particularly for spheroids with smaller radii.

Before we demonstrated the effectiveness of the AAfB technique for fabrication of tissues, we performed gel stability and spheroid viability experiments using 1.2% Carbopol and 0.5% alginate microparticles. Supplementary Fig. 3 demonstrated that Carbopol dissolved and flowed into the cell media substantially after 4 h of device preparation; however, the interface between the gel and media compartments in the case of the alginate microparticles was highly stable even after 24 h of the preparation of the device. In addition, Fig. 2f and Supplementary Fig. 4 showed that human mesenchymal stem cell (MSC) spheroids cultured in Carbopol had a reduced cell viability during a 3-day culture (~74% on Day 3; *p* = 0.0046), however, MSC spheroids maintained in alginate microparticles had ~93% cell viability on Day 3 (*p* = 0.7626). In addition, the removal of spheroids from Carbopol was more challenging than that from alginate microparticles due to leftover Carbopol residuals on spheroids. Therefore, we preferred to use 0.5% alginate microparticles for performing experiments pertaining cell viability and spheroid morphology as a response to different bioprinting parameters.

As it is known that external stressors might induce considerable damage to cell viability and spheroid shape^20,37^, we also performed experiments to explore the role of bioprinting parameters, including aspiration pressure and bioprinting speed, on spheroid deformation and viability. The results (Fig. 2g and Supplementary Fig. 5a) showed that increasing the bioprinting speed from 0.5 to 2.5 mm s^−1^ did not reduce the cell viability when the aspiration pressure was maintained constant. However, increasing the aspiration pressure from 70 to 170 mm Hg decreased the cell viability from ~95 to 68% (*p* = 0.0012) (Fig. 2h and Supplementary Fig. 5b). Figure 2i and Supplementary Fig. 5a showed that the increase in the bioprinting speed did not induce any significant change in the circularity of spheroids under a given aspiration pressure. On the other hand, increasing the aspiration pressure from 70 to 170 mm Hg increased the deformation of the spheroids and reduced the circularity from ~0.6 to 0.1 (*p* = 0.0008) (Fig. 2j and Supplementary Fig. 5b). As spheroids needed to be transferred rapidly in a safe manner without leading to significant deformations and impaired cell viability, we used 70 mm Hg aspiration pressure and 2.5 mm s^−1^ bioprinting speed throughout the entire study. While alginate microparticles were not as transparent as Carbopol (Supplementary Movie 1), we demonstrated the bioprinting of complex-shaped configurations using Carbopol and the bioprinting of tissues using the alginate microparticles.

### Applications of the AAfB technique.

**Fig. 3:**
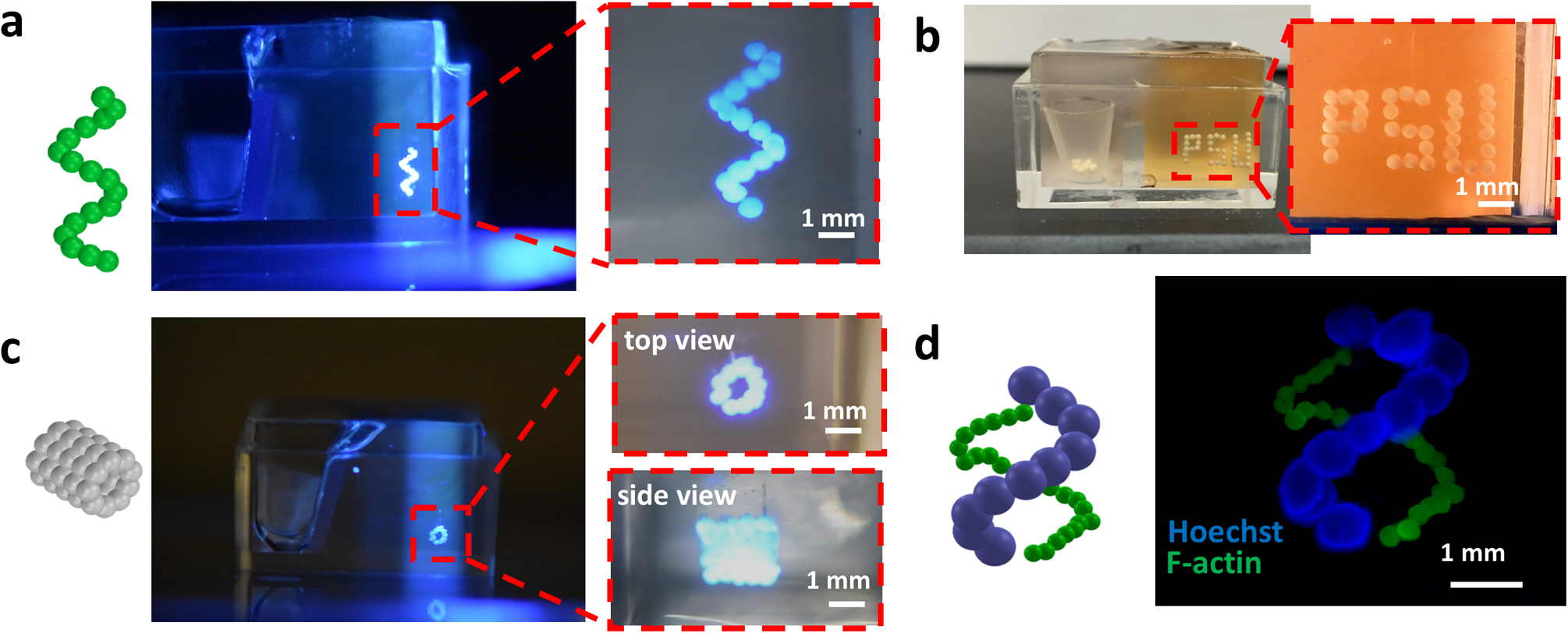
Aspiration-assisted freeform bioprinting of spheroids in different configurations. Schematic illustration and optical photographs of 3D bioprinted **a** helix-shape (mesenchymal stem cell (MSC) spheroids), **b** initials of Penn State University (PSU, MSC spheroids), **c** five-layer tubular (MSC spheroids), and **d** double helix-shape constructs using MSC spheroids with 150 μm (F-actin) and 450 μm (Hoechst) in radius in 1.2% Carbopol yield-stress gel. The red dashed line denotes the region magnified.

To demonstrate the potential of our AAfB technique, we demonstrated the bioprinting of a DNA-strand (Fig. 3a), of the acronym PSU for Penn State University (Fig. 3b and Supplementary Movie 2), and of five layers of circles forming a cylinder (Fig. 3c) using uniform size MSC spheroids (~175 μm in radius). We also bioprinted a double DNA-shaped strand MSC spheroids with different radii (150 and 450 μm) (Fig. 3d). In addition to complex-shaped configurations, we also demonstrated AAfB of cartilage- and bone-like tissue substitutes.

**Fig. 4:**
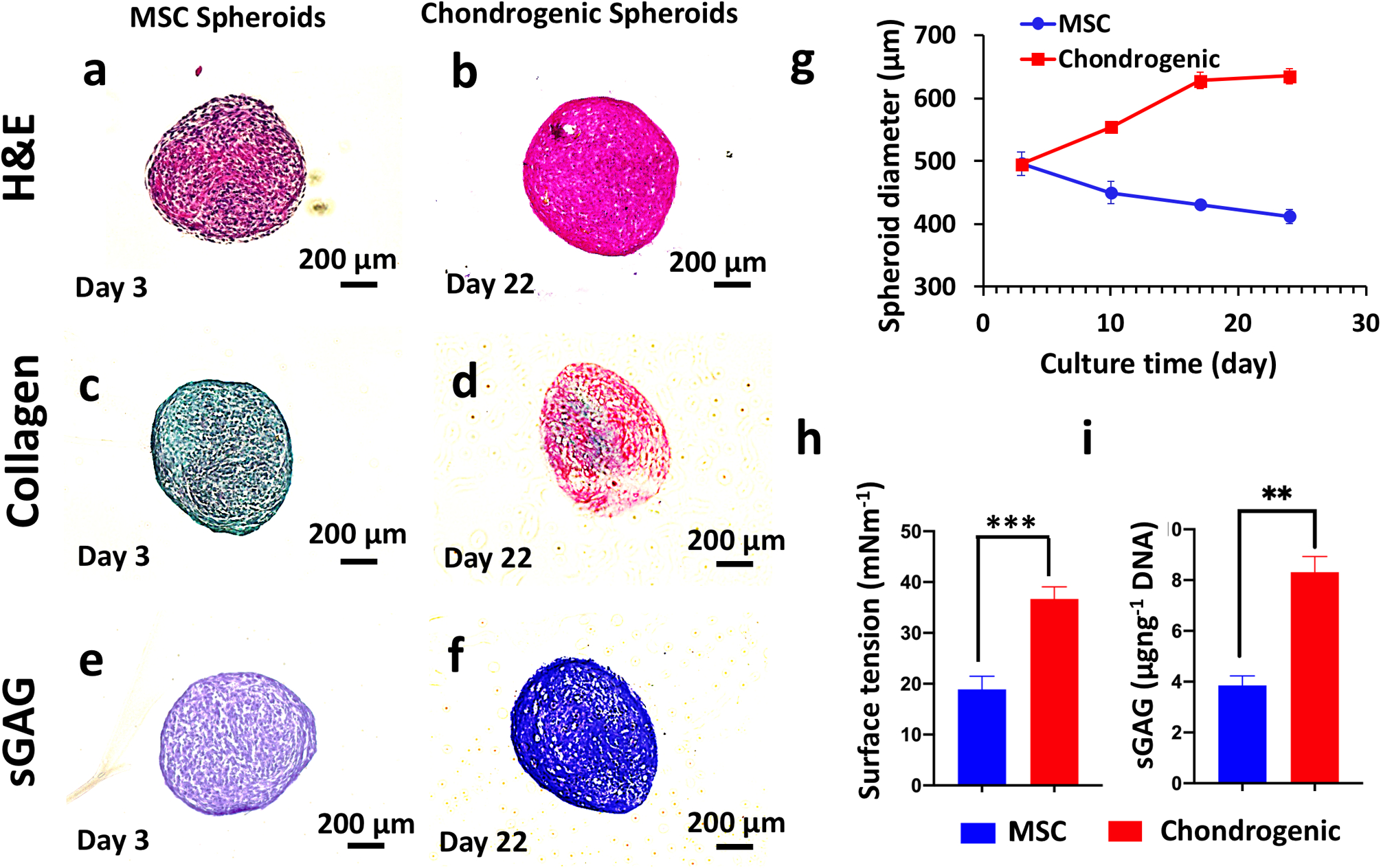
Chondrogenic differentiation of mesenchymal stem cell spheroids. Histological staining of mesenchymal stem cell (MSC) (at Day 3, prior to bioprinting for Strategy I) and chondrogenic spheroids (at Day 22, prior to bioprinting for Strategy II) **a**, **b** hematoxylin and eosin, **c**, **d** picrosirius red/fast green, and **e**, **f** toluidine blue staining. MSC spheroids were less dense and were negative (demonstrated by green color) for collagen and sGAG (demonstrated by purplish color), whereas the chondrogenic spheroids were denser and positive (demonstrated by green color) for collagen and sGAG (demonstrated by blue color). **g** Diameter change of MSC and chondrogenic spheroids over 24 days (*n* = 10). Note that chondrogenic spheroids were cultured in MSC growth media for the first 3 days of culture. The diameter of chondrogenic spheroids increased from 500 μm (on Day 3) to 600 μm (on Day 18) and retained their size for the remaining period of the culture until Day 24. The diameter of MSC spheroids gradually decreased from 500 μm (on Day 3) to 400 μm (on Day 24). **h** Surface tension, the surface tension values for both spheroids were within feasible ranges for bioprinting and **i** sGAG content measurements (normalized to DNA amount of MSC and chondrogenic spheroids at Day 24) (*n* = 3, **p < 0.01 and ***p < 0.001). A 2.2-fold increase in the sGAG content (μg ng^−1^ DNA) shown for chondrogenic spheroids as compared to MSC spheroids. Error bars were plotted as mean ± standard deviation.

Circular cartilage tissues were bioprinted using MSC spheroids following two strategies in order to investigate the effect of the chondrogenic differentiation timeline on the functional and structural properties of bioprinted tissues (Supplementary Fig. 6). In the first strategy, which we will refer to as Strategy I, MSC spheroids were maintained in the growth media for 3 days and then were 3D bioprinted into a circular shape on Day 3. The bioprinted constructs were removed from the gel on Day 4 and further maintained in a chondrogenic induction medium for 20 days. In the second strategy, which we will refer to as Strategy II, MSC spheroids were maintained in the growth medium for 3 days followed by a 19-day culture in a chondrogenic induction medium, and finally bioprinted on Day 22. Upon sufficient fusion, the bioprinted constructs were removed from the gel on Day 23 and samples were collected for further analysis on Day 24. Albeit detailed analysis of mechanical strength of the constructs fabricated by both strategies was out of scope in our current study, most of the bioprinted constructs demonstrated successful fusion, gaining sufficient mechanical handleability for removal from the gel. In order to understand the physical and biological properties of spheroids used in both strategies, we performed histological examinations of osteogenic and chondrogenic spheroids (Fig. 4a–f), size and surface tension measurement (Fig. 4g, h), and protein quantification (sulfated glycosaminoglycan (sGAG) content) (Fig. 4i).

**Fig. 5:**
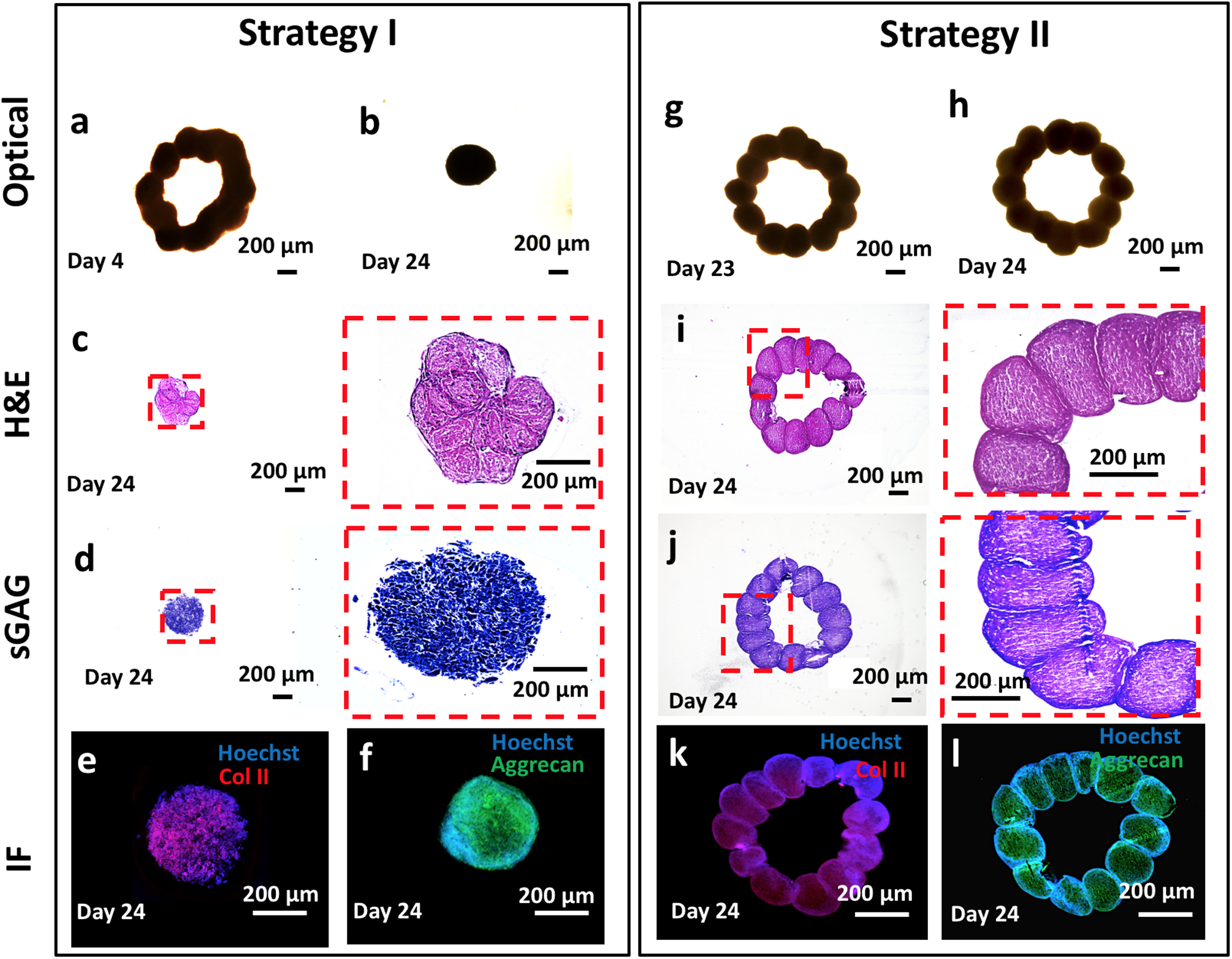
Aspiration-assisted freeform bioprinting of circular cartilage tissues. Strategy I: cartilages tissues were bioprinting using mesenchymal stem cell spheroids on Day 3 and removed from 0.5% alginate microparticles on Day 4. **a** A microscopic image showing a bioprinted construct after removal from alginate microparticles. **b** The final shape of the bioprinted cartilage at Day 24. Histological and immunostaining images of the bioprinted tissues at Day 24 including **c** hematoxylin and eosin (H&E), **d** toluidine blue, **e** Col-II, and **f** Aggrecan staining. Strategy II: cartilage tissues were bioprinted using chondrogenic spheroids at Day 22. **g** A microscopic image showing bioprinted construct after its removal from 0.5% alginate microparticles at Day 23. **h** The final shape of the bioprinted cartilage tissue at Day 24. Histological and immunostaining images of the bioprinted tissues at Day 24 including **i** H&E, **j** toluidine blue, **k** Col-II, and **l** Aggrecan. We showed that the bioprinted tissues in Strategy I exhibited chondrogenic properties; however, the bioprinted shape could not be retained because of the compaction of MSC spheroids. In Strategy II, we observed sufficient fusion between spheroids and the originally bioprinted circular arrangement was retained. The red dashed line denotes the region magnified.

We first investigated the differences in spheroids used for Strategy I (3-day culture in a growth medium) and Strategy II (3-day culture in a growth medium followed by a 19-day culture in a chondrogenic induction medium) prior to bioprinting in terms of hematoxylin and eosin (H&E) staining as well as collagen and sGAG content. MSC spheroids used for Strategy I were less dense (from H&E staining) and were negative for collagen and sGAG, whereas the spheroids used for Strategy II were larger in size, denser, and were positive for collagen and sGAG. Thus, in the rest of the study, we will refer to the spheroids used in Strategy I and Strategy II as MSC and chondrogenic, respectively. We also traced the change in spheroid size during the 24-day culture time (Fig. 4g). The diameter of chondrogenic spheroids increased from 500 μm (on Day 3) to a value slightly larger than 600 μm (on Day 18) and retained their size for the remaining period of the culture until Day 24. The diameter of MSC spheroids gradually decreased from 500 μm (on Day 3) to 400 μm (on Day 24). The surface tension is an important parameter that determines the structural integrity of the spheroids. The higher the surface tension the better the bioprinting was due to the spheroids’ decreased sensitivity to aspiration forces^20^. In this regard, chondrogenic spheroids had a surface tension that was approximately twice that of MSC spheroids (*p* = 0.000) (Fig. 4h). Furthermore, the surface tension values for both spheroids were within feasible ranges for bioprinting^20^. Finally, we also observed a 2.2-fold increase in the sGAG content (*p* = 0.002, μg ng^−1^ DNA) for chondrogenic spheroids as compared to MSC spheroids (Fig. 4i). We then used these spheroids from Strategy I and Strategy II to bioprint circular cartilage tissues, following the corresponding culture protocol for each strategy. The bioprinted circular shape was preserved during 1-day culture in the gel post-bioprinting and after removal of the tissue from the gel (Fig. 5a). However, the circular organization turned into a dense ball after 20 days of culture in the chondrogenic induction medium for Strategy I (Fig. 5b), whereas the circular shape was preserved in Strategy II. We performed H&E and sGAG staining as well as immunofluorescent (IF) staining for Type II collagen II (Col-II) and Aggrecan on bioprinted tissues on Day 24. In Strategy I, H&E staining showed compact arrangement of the bioprinted tissues (Fig. 5c). This finding was different from that revealed by the morphology and histology of MSC spheroids (Fig. 4a, b). In this case, sGAG deposition was noticed by positive sGAG staining (Fig. 5d). IF staining results showed that both Col-II and Aggrecan staining were positive (Fig. 5e, f). Here, we showed that the bioprinted tissues in Strategy I exhibited chondrogenic properties; however, the bioprinted shape could not be retained because of the compaction of MSC spheroids. In Strategy II, we observed that spheroids retained their shape, showed sufficient fusion between them, and retained original circular arrangements (Fig. 5g, h). H&E staining was similar to that of the chondrogenic spheroids (Fig. 5i). sGAG staining was positive, similarly to the case with chondrogenic spheroid (Fig. 5j). In addition, IF staining was positive to Col-II and Aggrecan (Fig. 5k, l).

We also demonstrated the bioprinting of bone tissue using osteogenic spheroids as building blocks. Osteogenic spheroids were fabricated in three different groups from MSC (Supplementary Fig. 7), and their differentiation was characterized in detail. In Group 1, MSC spheroids were formed on Day 0 and cultured in an osteogenic differentiation medium for 28 days. In Group 2, spheroids were formed after MSC were cultured on the tissue culture plate for 7 days, followed by an additional 21 days in osteogenic differentiation media. In Group 3, MSC were cultured on tissue culture plates in osteogenic differentiation media for 12 days before spheroids were formed. Spheroids were then cultured in an osteogenic differentiation medium for additional 16 days. For each group, spheroids were collected for analysis purposes on Days 14 and 28.

**Fig. 6:**
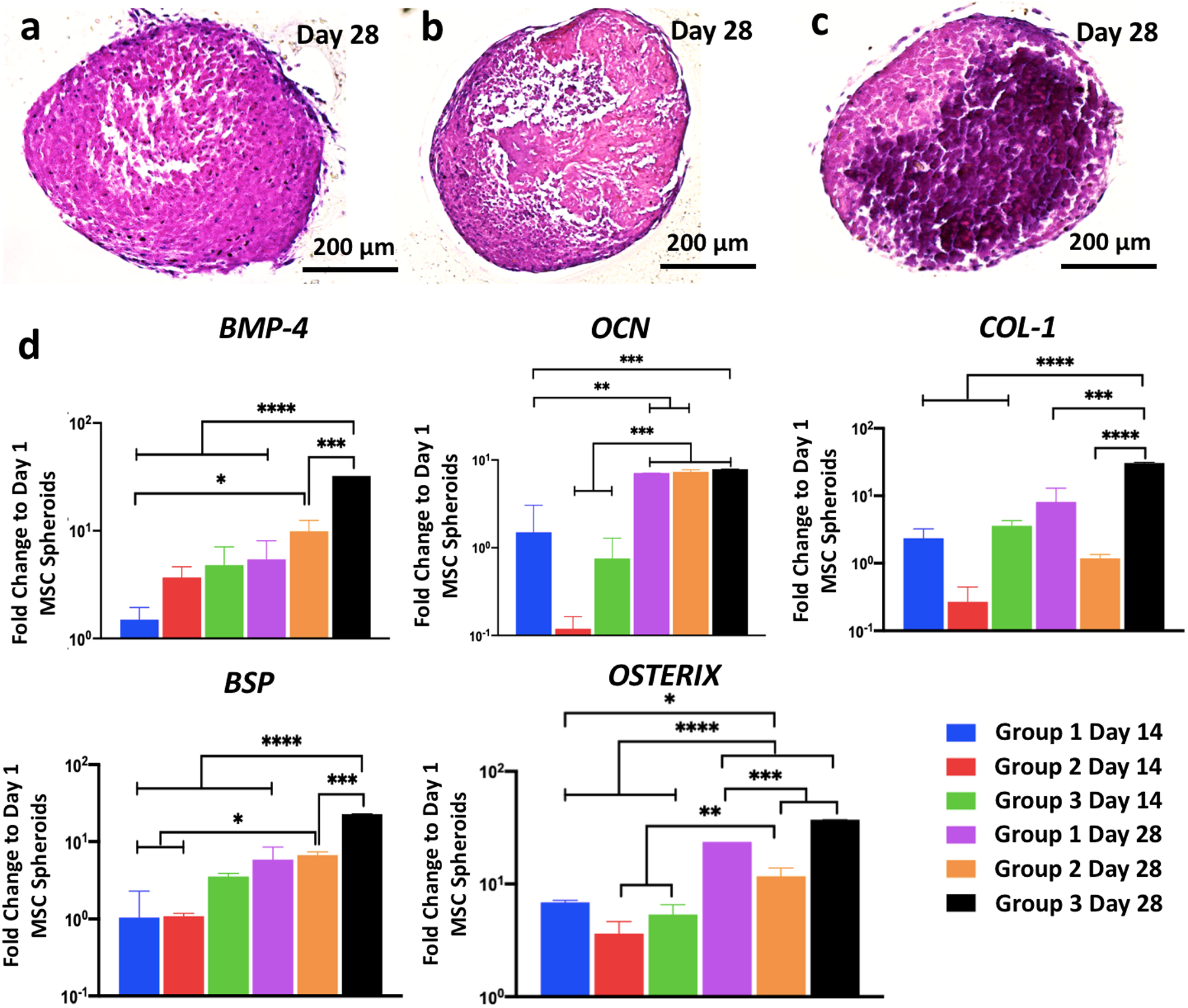
Osteogenic differentiation of mesenchymal stem cell spheroids. Hematoxylin and eosin staining images of spheroids of **a** Group 1, **b** Group 2, and **c** Group 3 at Day 28 demonstrating stronger bone matrix deposition in Group 3. **d** *BMP-4, OCN, COL-1, BSP*, and *OSTERIX* gene expressions of Group 1 Day 14, Group 2 Day 14, Group 3 Day 14, Group 1 Day 28, Group 2 Day 28, and Group 3 Day 28 (*n* = 3; *p < 0.05, **p < 0.01, ***p < 0.001, and ****p < 0.0001). All genes for all groups showed greater level of expression on Day 28 compared to Day 14. No significant difference was observed at Day 14 among groups; expression of *BMP-4* (4.8- and 32.3-folds), *COL-1* (3.6- and 30.5-folds), *BSP* (3.5- and 22.8-folds), and *OSTERIX* (5.3- and 37-folds) in Group 3 on Day 28 was significantly higher than those for Groups 1 and 2, respectively. Error bars have been plotted as mean ± standard deviation.

When the spheroids were compared on Day 28, H&E staining for Group 3 showed considerably more bone matrix deposition as compared to Groups 1 and 2 (Fig. 6a–c). Confocal images of Group 3 demonstrated the strongest expression of OSTERIX, which is a late-stage osteogenic differentiation marker (Supplementary Fig. 8). Expression of osteogenic genes was investigated for different groups of spheroids, including bone morphogenetic protein-4 (*BMP-4*), osteocalcin (*OCN*), Type I collagen (*COL-1*), bone sialoprotein (*BSP*), and *OSTERIX*, at Days 14 and 28 (Fig. 6d). Overall, all genes for all groups showed greater level of expression on Day 28 as compared to Day 14. Although gene expressions on Day 14 exhibited no significant difference among groups, expression of *BMP-4* (4.8- (*p* < 0.0001) and 32.3- (*p* = 0.0001) folds), *COL-1* (3.6- (*p* = 0.0003) and 30.5- (*p* < 0.0001) folds), *BSP* (3.5- (*p* < 0.0001) and 22.8- (*p* = 0.0001) folds), and *OSTERIX* (5.3- (*p* = 0.0001) and 37- (*p* < 0.0001) folds) in Group 3 on Day 28 was significantly higher than those for Groups 1 and 2, respectively.

For bioprinting of bone tissues, we followed three strategies in order to understand the role of the osteogenic induction timeline on the formation of bone tissue (Supplementary Fig. 9). In Strategy I, Group 1 Day 14 osteogenic spheroids were used (i.e., MSC spheroids that were formed at Day 0 and then cultured in osteogenic differentiation media for 14 days). Group 1 spheroids were bioprinted on Day 14 and the tissue was removed from the gel on Day 15, and cultured in osteogenic induction media for 13 days, completing a 28-day period in total. In Strategy II, Group 2 osteogenic spheroids were used (spheroids formed after 7-day 2D differentiation followed by 7-day 3D differentiation). Spheroids were bioprinted on Day 14 and the bioprinted tissues were removed from the gel after spheroids fused each other sufficiently on Day 15, followed by 13 days of culture in an osteogenic induction medium. Finally, Strategy III utilized Group 3 osteogenic spheroids (spheroids formed after 12-day 2D differentiation followed by 2-day 3D differentiation). In this group, bioprinted tissues were cultured in the osteogenic differentiation media for 13 days after removal from the gel.

**Fig. 7:**
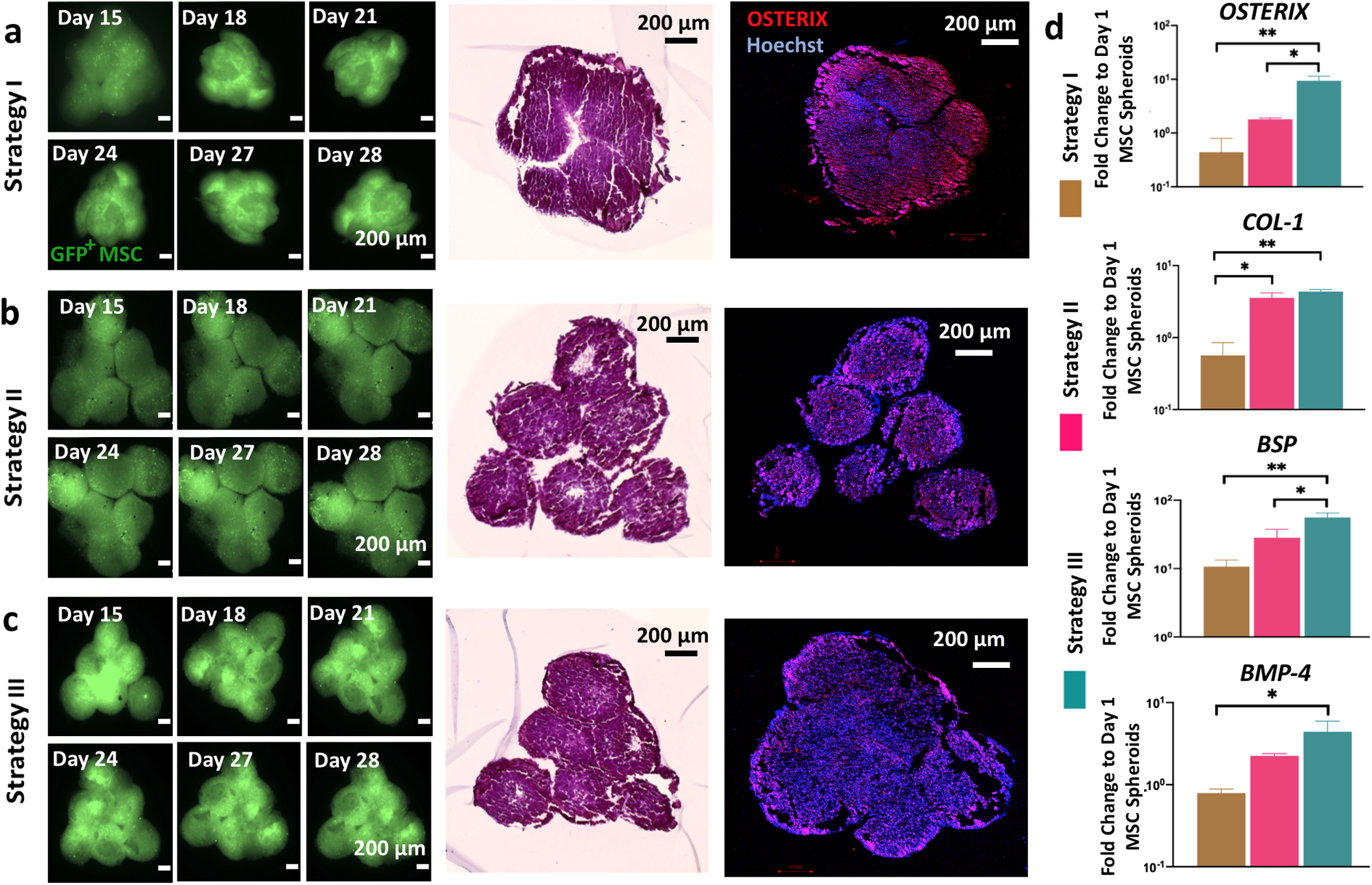
Aspiration-assisted freeform bioprinting of osteogenic tissues in a yield-stress gel. Time-lapse images of green fluorescent protein (GFP)^+^ mesenchymal stem cell (MSC) spheroids and their immunostaining (Hoechst in blue and OSTERIX in red) and hematoxylin and eosin staining for bioprinted bone tissue using **a** Strategy I, **b** Strategy II, and **c** Strategy III. In Strategy I, the original shape could not be conserved due to compaction, whereas in Strategies II and III, the triangle shape was well preserved. **d** *OSTERIX, COL-1, BSP*, and *BMP-4* gene expressions of 3D bioprinted bone tissues cultured using different strategies (*n* = 3; *p < 0.05, **p < 0.01, and ***p < 0.001). Constructs in Strategy III exhibited the highest expression level for all genes—23.5- and 5.2-fold increase for *OSTERIX*, 7.9- and 1.2-fold increase for *COL-1*, 5.2- and 2-fold increase for *BSP*, and 5.5- and 2-fold increase for *BMP-4*, respectively, compared to Strategies I and II. Error bars have been plotted as mean ± standard deviation.

We bioprinted triangle-shaped osteogenic tissues using six spheroids each following these three strategies. Fluorescent images of the tissues (at Days 15, 18, 21, 24, 27, and 28) were used to qualitatively observe the shape changes due to the fusion and compaction of green fluorescent protein (GFP^+^) MSCs (Fig. 7a–c). In Strategy I, the original shape could not be conserved due to compaction, whereas in Strategies II and III, the triangle shape was well preserved. H&E staining also corroborated this finding by demonstrating more shape retention (more triangular) in the tissues bioprinted with Strategies II and III as compared to Strategy I. IF images showed that OSTERIX staining was more uniformly expressed for Strategies II and III but weakly expressed in the core of bone tissues bioprinted with Strategy I (Fig. 7a–c).

Expression of osteogenic genes, including *OSTERIX*, *COL-1*, *BSP*, and *BMP-4*, was also evaluated (Fig. 7d). Constructs in Strategy III exhibited the highest expression level for all genes than those in Strategies I and II, namely, 23.5- (*p* = 0.0099) and 5.2- (*p* = 0.0157) fold increase for *OSTERIX*, 7.9- (*p* = 0.0063) and 1.2- (*p* = 0.2943) fold increase for *COL-1*, 5.2- (*p* = 0.0042) and 2- (*p* = 0.0319) fold increase for *BSP*, and 5.5- (*p* = 0.0146) and 2- (*p* = 0.0981) fold increase for *BMP-4*, respectively. In addition, the expression level of *OSTERIX* (4.5-fold increase; *p* =0.5575), *COL-1* (6.4-fold increase; *p* = 0.0123), *BSP* (2.6-fold increase; *p* = 0.1027), and *BMP-4* (2.8-fold increase; *p* = 0.2142) was higher in Strategy II as compared to those in Strategy I. Our results indicate that the longer the cells were exposed to induction media on 2D, the more pronounced the osteogenic differentiation in spheroids as well as bioprinted tissues.

## Discussion

Although extrusion-based bioprinting in yield-stress gels has already been demonstrated in the literature^5,6,10,14,17,19,38^, its utilization in bioprinting of prefabricated cellular aggregates is quite challenging. Here, we presented a bioprinting approach with the ability to bioprint cellular aggregates such as tissue spheroids in an accurate and precise manner in 3D. In this study, the presented AAfB approach enabled the freeform biofabrication of 3D complex-shaped constructs using spheroids as building blocks: we want to stress that this is not similarly achievable using existing bioprinting methods^25,26,28^. In addition to its potential in precise positioning of spheroids in 3D, the AAfB approach also made it feasible to bioprint spheroids with radii ranging from 150 to 450 μm (Fig. 3a–d).

Bioprinting positional accuracy increased with the concentration of Carbopol. Due to the shear thinning behavior of the yield-stress gel, when the Carbopol concentration was low (e.g., 0.8%), the yield-stress gel liquefied and maintained insufficient viscosity and self-healing properties to hold the bioprinted spheroids in place accurately. The positional accuracy was increased with increasing Carbopol concentration. However, higher levels of aspiration pressure were required in these cases to transfer the spheroids from their initial location to their final placement. The higher aspiration pressure might induce substantial spheroid damage, such as their breakage during transition into the gel or their complete aspiration into the nozzle. Consequently, to exploit the potential of this technique, it was crucial to determine of optimal gel properties and bioprinting speeds to guarantee the spheroids’ accurate placement while preserving their integrity and viability. Thus, Carbopol concentration of 1.2% was preferred for 3D patterning of spheroids. While it might be convenient to assume that the gel properties were uniform within the entire gel domain, our empirical observation was that the cell medium diffused into the gel and changed the gel properties accordingly for Carbopol. Carbopol started to dissolve and flow into media compartment after 4 h. On the other hand, alginate microparticles exhibited structural integrity, where the gel properties were highly uniform over 24 h (Supplementary Fig. 3). Thus, alginate microparticles can be considered more promising for prolonged bioprinting processes. Clearly, the bioprinting time could be minimized by increasing the bioprinting speed. However, a substantial increase in the bioprinting speed could also result in failure as spheroids could easily get stuck at the medium/gel interface due to the substantial resistance exerted by the gel (Supplementary Movie 3). In addition, decreasing the speed did not improve the cell viability while maintaining the aspiration pressure the same; however, increasing the aspiration pressure resulted in significant cell death and deformation in spheroids. As increasing the bioprinting speed in the gel domain required higher aspiration pressure due to the increased drag force, we used 2.5 mm s^−1^ as our preferred bioprinting speed, which allowed for a rapid enough assembly of the presented tissue models while remaining safe enough to successfully transfer the spheroids from the cell media to the gel.

After bioprinting, the yield-stress gels were trimmed in order to increase the amount of media and the exposure of spheroids to the media to enhance the viability of spheroids (Supplementary Fig. 10). We experienced significant decrease in the viability of spheroids in Carbopol (from ~93 to 74% (*p* = 0.0046) in 3 days, Fig. 2f), which could be due to the biological inertness of the Carbopol as well as the possible risk of pH changes (from 7.4 to 6.9 in our study)^13,19,39^. In addition, the properties of Carbopol changed due to dissolution of Carbopol in the media. As a result, bioprinted cartilage and bone tissues dissembled quite a few times during their removal from Carbopol in our preliminary efforts (<40% efficiency). In general, controlling the pH level of Carbopol and removing the fused spheroids from the Carbopol were not trivial^5,15,19^. Thus, alginate microparticles were preferred for bioprinting of cartilage and bone tissues, where the cell viability was maintained at ~93% despite 3 days of incubation. In addition, alginate microparticles were very stable during prolonged bioprinting and the removal of spheroids was more straightforward with the use of alginate lyase (Supplementary Fig. 11). Thus, alginate microparticles can be considered a promising yield-stress gel for AAfB purposes. However, we still observed disassembly of bioprinted constructs while removal from alginate microparticles but the efficiency of successfully removing the bioprinted constructs from alginate microparticles (>65%) was much higher than that from Carbopol. This could be due to the entrapment of alginate microparticles between spheroids, which can be improved by further decreasing the size of alginate microparticles or increasing the tissue culture time in the gel as such an issue can be problematic when bioprinting scalable highly intricated geometries. In addition, the transparency of alginate microparticles was limited compared to that of Carbopol, which should be improved for building more advanced automated platforms with image recognition features. Nevertheless, synthesis and development of novel yield-stress gels, possessing optimal mechanical properties in terms of yield stress and shear thinning as well as additional physical properties such as self-healing, transparency, biocompatibility, and the ability to be drained from the bioprinted tissue without harming their integrity, will greatly improve the deployment of platforms for fabrication of scalable human tissues and organs at the clinically relevant volumes. Indeed, a guest-host yield-stress gel (hyaluronic acid), which was demonstrated in 3D printing of vascular channels^40^, is currently being investigated for bioprinting of a cardiac disease model by Burdick et al.^41^.

The theoretical estimation of the force exerted on the spheroid by its environment during bioprinting was elementary and meant to capture how the nozzle aspiration pressure scales with the bioprinting speed and the spheroid’s radius. Our model was particularly limited in capturing the details of the spheroid transfer through the medium/gel interface where the elastic and plastic behavior of the gel both significantly contribute to the force on the spheroid. Finally, our modeling did not include any considerations on the deformability of the spheroids, which is of primary concern when assessing viability. The future enhancement of the proposed bioprinting technique, especially when trying to tune the gel’s physical properties to achieve an optimal bioprinting accuracy and viability, can benefit from a more sophisticated analysis of spheroid motion mechanics.

In our attempts preceding this study, we aspirated and lifted spheroids using a glass pipette with a radius of about 40 μm (see our recently published work^20^). We encountered problems in the use of a pipette when we transitioned spheroids in the gel domain, i.e., as we crossed the medium/gel interface. As depicted in Supplementary Movie 4, spheroids were prone to bounce at the pipette tip because of insufficient aspiration forces against the drag force, which was due to the reduced exposure area of aspiration. In addition, as the pipette enlarged significantly toward its upper portion, we observed other issues such as substantial damages to the gel along with slower and diminished healing. Because of these reasons, we switched to metallic straight nozzles with a larger nozzle radius (inner radius of 100 μm). As long as we bioprinted spheroids with a radius of at least 150 μm at least, the metallic straight nozzles proved sufficient to perform the presented bioprinting work. This said, smaller nozzle tips or even pipette tips could still be utilized for bioprinting of spheroids with radii smaller than 50 μm. In addition, the metallic straight nozzles did not induce any major deformations at the interface as both Carbopol and alginate microparticles had sufficient self-healing property to recover the damaged region caused by the back and forth motion of the nozzle. Bioprinting of the constructs was also performed further away from the interface; thus, minor damages at the interface did not affect our bioprinting capabilities. For biofabrication of scalable constructs, the nozzle could be controlled to penetrate into the gel from different regions at the interface, allowing sufficient time for the self-healing of the gel and eventually reducing the deformation at a particular point.

Here, the spheroids were bioprinted into a circular arrangement using two different strategies in order to understand the role of MSC or chondrogenic spheroids in successful bioprinting of cartilage tissues. For both the cases, the spheroids were placed physically in contact with each other to facilitate fusion without the need of any external support. We identified considerable differences between MSC and chondrogenic spheroids in term of biological, structural, and mechanical properties. In particular, MSC spheroids shrank in size while chondrogenic spheroids grew over time, which could be due to the significant deposition of chondrogenesis-related extracellular-matrix deposition, which, in turn, yielded higher surface tension and sGAG content in chondrogenic spheroids^21^. Bioprinting of chondrogenically differentiated spheroids generated tissues with improved chondrogenic properties and shape fidelity. We cultured the cartilage tissue for a short period of time post-bioprinting. Although further compaction could be expected overtime, we here successfully demonstrated minimal compaction with sufficient structural integrity over the entire course of differentiation using Strategy II. The resultant tissue could be implanted in vivo (i.e., cartilage rings around bronchus) or further cultured in vitro with a confining mechanical support (such as a rod inside its lumen) in order to prevent further compaction.

In bioprinting of osteogenic spheroids, three different strategies were designed to study the role of monolayer versus 3D induction on successful formation of bone tissue with controlled morphology. The results indicated that longer culture period in monolayer improved the construct fidelity as evidenced by the result of Strategy III (Fig. 7c). This could be due to the increase exposure of MSC to osteogenic differentiation media or improved osteogenesis of MSC due to the substrate stiffness of tissue culture plate^42,43^. As MSC in 3D spheroid culture had limited integrin-mediated adhesion with respect to tissue culture plates, we observed enhanced bone formation at the gene and protein level^42^. It is also known that, osteogenically differentiated MSC could have limited proliferation, which might reduce the fusion and compaction of osteogenic spheroids in bioprinted bone tissues. Although we attempted to optimize the compaction of spheroids using different culture strategies, other approaches can also be tested, such as inhibition of collagen as it is known that collagen synthesis in spheroids plays a role in spheroid compaction^44,45^. However, compaction-related shape change is an inherent problem in scaffold-free systems. Therefore, one can prefer scaffold-based systems via 3D printing of polymeric scaffolds or cell-laden hydrogels when shape fidelity and preservation is a priority.

Here, to demonstrate the proof-of-concept of the newly developed AAfB approach, we fabricated two geometrical configurations—triangular and circular for bone and cartilage, respectively. Nevertheless, this approach can be used with slight modifications to easily reconfigure anatomically correct shapes of tissues for transplantation purposes. Despite we demonstrated a technology with picking and placing spheroids one by one, high-throughput systems are required in order to build clinically relevant volumes of tissues. For example, with the use of current setup, 1 cm^3^ tissue can be bioprinted in about 6 days; however, with the use of high throughput and more advanced automation systems, the bioprinting process could be shortened to a few hours. To bioprint scalable tissue constructs, integration of vascularization is also very critical^46^. In this regard, the spheroids could be bioprinted into functional gels (such as fibrin and collagen) with perfusable vascular networks, where angiogenic capillary sprouting can be induced to facilitate the anastomosis between these sprouts and the perfusable vascular networks. Although we demonstrated tissues with isotropic structure, some tissues may require anisotropy such as articular cartilage, muscle, etc. In this regard, instead of using spheroids with uniform and isotropic properties, mini-tissue building blocks with anisotropic properties can be utilized to reconstitute the anisotropic organization of these tissues.

In summary, we presented a highly effective approach in 3D bioprinting and positioning of tissue spheroids by explaining the interplay between the bioprinting process and yield-stress gel properties. Such a platform enabled us to pattern tissue spheroids in 3D, which will have tremendous applications such as, but not limited to, tissue engineering and regenerative medicine, disease modeling, drug screening, and biophysics.

## Materials and methods

### Preparation of the yield-stress gels.

#### Carbopol preparation:

0.8, 1.2, and 1.6% (w v^−1^) Carbopol ETD 2020 NF (Lubrizol Corporation, OH) was dispersed in RoosterBasal™ MSC media (RoosterBio Inc., MD) under sterile conditions. NaOH was added dropwise to the Carbopol-dispersed gel to adjust the pH to 7.4, which facilitated the maximum swelling of Carbopol and its biocompatibility. The GH was then homogenized using a vortex blender for 20 min, centrifuged at 1000 × *g* for 15 min, and incubated at 37 °C and 5% CO_2_ before further use.

#### Preparation of alginate microparticles:

To prepare alginate microparticles, all equipment was sterilized with 70% ethanol and ultraviolet light 30 min. 0.5 g sodium alginate was dissolved in 100 ml Dulbecco’s Modified Eagle’s Medium (DMEM) in a sterile environment. The solution, hence made, was loaded into a syringe and dispensed into 4% CaCl_2_ bath. After crosslinking of alginate for 30 min, fibers were collected, washed thrice with DMEM, and blended at 465 × *g* for 10 min to obtain alginate microparticles. The resultant microparticles were then divided 50 ml conical tubes and centrifuged at 2000 × *g* for 5 min. Next, the microparticles were purified and washed thrice by consecutive discarding and replacing chondrogenic or osteogenic differentiation media (Cell Applications, CA) to remove any undissolved particles. Alginate particles were then placed to cover only half area of square Petri dishes and the remaining part was filled with the respective chondrogenic or osteogenic media. Safranin O stain kit (American MaterTech Scientific) was used according to the manufacturer’s instructions to visualize microparticles, and imaged using a microscope (EVOS, Thermo Fisher Scientific). The particle size was quantified by laser granulometry using a Mastersizer 3000 from Malvern PANalyticals (MA).

### Rheological analysis.

Rheological measurements of the yield-stress gels were performed using an MCR 302 rheometer (Anton Paar, VA) using a 25 mm diameter parallel-plate geometry. A Peltier system was employed for temperature control. Amplitude tests were applied to determine viscous and elastic properties of gels at a constant frequency of 1 Hz and a strain range from 0.01 to 100% at a constant temperature of 25 °C. Frequency sweep test was carried out to determine storage modulus (G′) and loss modulus (G″) and complex viscosity (η*) at a frequency ranging from 0.1 to 100 rad s^−1^ at a strain of 5% for 1.6% Carbopol and a strain of 1% for 0.8 and 1.2% Carbopol and 0.5% alginate microparticles, which were within the linear viscoelastic range of each gel.

### Gel stability test.

Carbopol and alginate microparticles were loaded into the gel compartment of the device and the remaining area was filled with tissue-specific media. Gel and media loaded devices were placed in front of a USB camera (USB2-MICRO-250X, Plugable, China) and a snapshot was taken every 4 h for 12 h. The side and top view micrographs at 24 h were taken using a Nikon D7200 camera (Nikon, Tokyo, Japan).

### Fabrication and differentiation of MSCs spheroids.

Human MSCs (RoosterBio Inc.) and GFP-labeled MSCs (GFP^+^ MSCs) (Cyagen, CA) were used in experiments. Both types of MSCs were cultured in RoosterBasal™ MSC medium, composed of RoosterBooster™ MSC-XF growth supplement, 100 U mL^−1^ penicillin, 100 μg mL^−1^ streptomycin, and 1 μg mL^−1^ fungizone (Life Technologies, CA), under a humidified atmosphere with 5% CO_2_ at 37 °C. To prepare spheroids, MSCs were trypsinized and centrifuged to form cell pellets. 200 μL of the cell suspension (1 × 10^5^ and 2.5 × 10^5^ cells per mL for osteogenic and chondrogenic spheroids, respectively) was transferred into each well of a 96-well plate (Greiner Bio One, NC) to obtain 2 × 10^4^ and 5 × 10^4^ cells per well for osteogenic and chondrogenic spheroids, respectively. Cells were cultured in MSC growth media during spheroid formation and the medium was changed every 3 days. MSC spheroids were differentiated into chondrogenic and osteogenic lineages for different applications using human chondrocyte and osteoblast differentiation media^47^.

### AAfB process.

For the bioprinting setup, we utilized our AAB system^20^. A square Petri dish was used as a device to hold the gel and cell culture media (Fig. 1a), where a polydimethylsiloxane slice was utilized to separate both compartments in order to obtain a vertically oriented interface. Spheroids were placed in a reservoir, which was submerged in the tissue-specific media. When the reservoir was transferred into the Petri dish, tissue-specific cell media was filled to cover the remaining area in the Petri dish. A 27G needle (Nordson, OH) was used to pick the spheroids from the reservoir and transfer them from cell culture media into the yield-stress gel with a speed of 2.5 mm s^−1^. Two microscopic cameras (one for each side of the Petri dish) were used to visualize the bioprinting process in real time. In order to validate the theoretical results, MSC spheroids with a wide range of radius (from 150 to 400 μm) were bioprinted into 1.2% Carbopol and 0.5% alginate microparticles.

### Accuracy and precision measurement of bioprinting.

In order to investigate the effect of the yield-stress gels on the positional accuracy and precision, MSC spheroids were bioprinted at predetermined target positions in 0.8, 1.2, and 1.6% Carbopol and 0.5% alginate microparticles^20^. A calibration slide (Motic, China) was placed at the bottom of the Petri dish to monitor the target position. After spheroid deposition, images of the bioprinted spheroids (*n* = 5) were taken using cameras under the calibration slide and analyzed using ImageJ. Accuracy was represented as the root mean square error (RMSE), which was calculated using the equation as below:

(7) $$\text{RMSE}={\left[\left[\sum_{i=1}^{n}{\left(X_{\text{target}}-X_{i}\right)}^{2}+{\left(Y_{\text{target}}-Y_{i}\right)}^{2}\right]/n\right]}^{1/2},$$

where *X*_target_ and *Y*_target_ represent *X* and *Y* coordinates of the target position, respectively, *X*_*i*_ and *Y*_*i*_ are the position of the measured values in *X*- and *Y*-axis, respectively, and *n* is the sample size. Precision was represented as the square root of the standard deviation.

### Cell viability analysis.

MSC spheroids were loaded into 1.2% Carbopol and 0.5% alginate microparticles using a pipette. The media was removed, and the gel was trimmed from the ends to carefully preserve the structure of loaded spheroids (Supplementary Fig. 10). For Carbopol, the spheroids were removed and rinsed with PBS several times. On the other hand, spheroids loaded in alginate microparticles were transferred to another sterile Petri dish. 3 ml sodium citrate (4% w v^−1^ in PBS) solution was added and pipetted gently for 10 min in order to dissolve alginate particles (Supplementary Fig. 11). Next, spheroids were washed thrice with PBS and cell viability was evaluated at Days 1, 2, and 3 after culture. Spheroids were incubated in a cocktail mixture comprising of 1 μM calcein AM and 1.6 μM ethidium homodimer-1 (Life Technologies, NY) in PBS for 30 min, in which live cells were stained in green, while dead cells were stained in red. Z-stack images were taken on the EVOS microscope. ImageJ (National Institutes of Health, MD) was used for quantitative analysis for red and green fluorescent intensity to quantify cell viability^21,48^.

### Interaction between bioprinting parameters, spheroid viability, and deformation.

The effects of bioprinting parameters, including bioprinting speed (0.5, 1.5, and 2.5 mm s^−1^) and aspiration pressure (70, 120, and 170 mm Hg), on cell viability were analyzed through fluorescent LIVE/DEAD staining as discussed before (see “Cell viability analysis”). The stained spheroids were imaged by fluorescence microscopy (Axiozoom, Zeiss, Germany). Each image was then analyzed using ImageJ.

The circularity of bioprinted spheroids at different bioprinting speeds (0.5, 1.5, and 2.5 mm s^−1^) and aspiration pressure levels (70, 120, and 170 mm Hg) was also analyzed using ImageJ software, and calculated based on the following equation below:

(8) $$C=4\pi \times \frac{A}{P^{2}},$$

where *C* is the circularity, *A* is the area, and *P* is the perimeter of a spheroid. The value of circularity has a range of 0 (infinitely elongated polygon) to 1 (perfect circle).

### Physical properties of MSC and chondrogenic spheroids.

The chondrogenic differentiation of MSC spheroids was started on Day 3 and the diameter of spheroids were measured by the EVOS microscope until Day 24. Surface tension of spheroids was also measured by a micropipette aspiration technique according to the protocol established in our lab^21,48^. Customized straight micropipettes (~40 μm in radius), fabricated from glass pipettes (VWR, PA) using a P2000 Flaming/Brown micropipette puller (Sutter Instrument, CA), were used to aspirate spheroids. The aspirated spheroids were monitored via a STC-MC33USB monochromatic camera (Sentech, Japan), appended with 1–61448 and 1–61449 adapter tubes (Navitar, Rochester, NY). Surface tension of the MSC and chondrogenic spheroids was then calculated from the data on Day 24.

### Histological analysis of spheroids.

MSC and chondrogenic spheroids were fixed with 4% paraformaldehyde and sectioned with paraffin embedding to obtain 10 μm sections. H&E staining was performed on the sections using Leica Autostainer XL (Leica, Germany). For sGAG visualization using Toluidine Blue O staining, sections were incubated in a Toluidine Blue solution (0.1% in DI water, Sigma Aldrich, MO) at room temperature for 2 min. The dye was then removed and samples were washed twice with DI water, followed by dehydration with ascending alcohol and clearing with xylene. All samples were mounted and imaged using the EVOS microscope.

### Evaluation of sGAG content.

sGAG content was determined by DMMB dye-binding assay^21^. Briefly, MSC and chondrogenic spheroids were washed and digested in 500 μL solution of 0.1 mg mL^−1^ papain extraction reagent at 65 °C in water bath for 18 h. 20 μL of the digested samples was mixed with 200 μL DMMB solution and the absorbance was measured at 525 nm using a microplate reader (PowerWaveX, BioTek, Winooski, VT). Serially diluted solution of chondroitin 4 sulfate was prepared as the standard and the sGAG content was calculated according to the standard curve. The DNA content of same samples was also measured using the Quant-iT™ PicoGreen dsDNA Assay Kit (Molecular Probes Inc., Eugene, OR) according to the manufacturer’s instructions. Fluorescence intensity was determined by a SpectraMax multidetection microplate reader (Molecular Devices, Inc., Sunnyvale, CA) using a wavelength of 480 nm (excitation) and 520 nm (emission). sGAG content from each sample was normalized to dsDNA content.

### Bioprinting of cartilage tissue.

In order to optimize bioprinted cartilage tissue, two strategies were designed (Supplementary Fig. 6). In Strategy I, MSC spheroids on Day 3 were used for bioprinting, and the bioprinted constructs were incubated under chondrogenic differentiation for another 21 days. In Strategy II, MSC spheroids were cultured with chondrogenic media for 19 days, followed by bioprinting and cultured for 2 days in the form of constructs. After bioprinting of chondrogenic spheroids, the excess amount of gel was gently removed (without affecting the structural integrity of the bioprinted constructs) in order to maximize the diffusion of cell media to better support the growth of the tissue, as explained in section “Cell viability analysis.” The constructs obtained by two strategies were characterized by H&E staining and Toluidine Blue O staining as described in section “Histological analysis of spheroids” to visualize the tissue morphology and chondrogenesis.

### Immunohistochemistry of the bioprinted cartilage tissues.

Primary monoclonal antibodies were purchased from Abcam (MA) and fluorescence-conjugated secondary antibodies were purchased from Life Technologies (CA). Sections of MSC and chondrogenic spheroids were treated using Triton-X 100 (0.1% in PBS) for 10 min and blocked with normal goat serum (NGS, 10% in PBS) for 1 h. Samples were then incubated with monoclonal rabbit anti-human Col-II (1:200), mouse anti-human aggrecan (1:50), and NGS (negative control) overnight at 4 °C, respectively. Samples were washed twice with PBS and incubated using secondary antibodies (goat anti-rabbit IgG (H + L)-Alexa Fluor 647 for Col-II, and goat anti-mouse IgG (H + L)-Alexa Fluor 488 for aggrecan, 1:200) for 1 h. Samples were also incubated with Hoechst 33258 (1:200) for 10 min. Images for each marker were taken using a Zeiss Axiozoom microscope (Carl Zeiss Microscopy LLC, Germany).

### Gene expression of osteogenic spheroids using real time polymerase chain reaction (RT-qPCR).

In order to investigate the effect of different strategies on the osteogenesis of spheroids, three groups were designed (Supplementary Fig. 7). In Group 1, spheroids were prepared from MSCs and cultured for 28 days in osteogenic differentiation media. In Group 2, MSCs were cultured in monolayer for 7 days, followed by fabricating and culturing spheroids for 21 days in osteogenic differentiation media. In Group 3, MSCs were cultured in monolayer for 12 days, followed by fabricating and culturing spheroids for 16 days in osteogenic induction media. For all groups, the total induction period in monolayer culture and in the form of spheroids was kept 28 days in total.

For testing of bone-specific gene expression using RT-qPCR, single differentiated spheroids per sample were homogenized in TRIzol reagent (Life Technologies, CA), followed by adding 0.2 mL chloroform per 1 mL TRIzol reagent and centrifuging the mixture at 12,000 × *g* for 15 min at 4 °C. The upper aqueous phase with RNA was transferred and RNA was then precipitated by adding 0.5 mL isopropyl alcohol per 1 mL TRIzol reagent, followed by centrifuging at 12,000 × *g* for 10 min at 4 °C. Subsequently, the precipitated RNA was rinsed twice by 75% ethanol, air-dried for 10 min, and dissolved in 50 μL diethyl pyrocarbonate-treated water. RNA concentration was measured using a Nanodrop (Thermo Fisher Scientific, PA). Reverse transcription was performed using AccuPower® CycleScript RT PreMix (BIONEER, Korea) following the manufacturer’s instructions. Gene expression was analyzed quantitatively with SYBR Green (Thermo Fisher Scientific, PA) using a QuantStudio 3 PCR system (Thermo Fisher Scientific). Bone-specific genes tested included *OSTERIX* (Transcription factor Sp7), *COL-1*, *OCN*, *BMP-4*, and *BSP*. The reader is refereed to Table 1 for the gene sequences. Expression levels for each gene were then normalized to glyceraldehyde 3-phosphate dehydrogenase (*GAPDH*). The fold change of MSC spheroids after formation on Day 2 was set as onefold and values in osteogenic groups were normalized with respect to the control.

### Bioprinting of osteogenic tissues.

In order to investigate the effect of different culture strategies on formation of bone tissue, three strategies were designed. In Strategy I, spheroids were prepared using MSCs and cultured with osteogenic induction media for 14 days. In Strategy II, MSCs were cultured with osteogenic induction in monolayer for 7 days, followed by fabricating and culturing spheroids for 7 days with osteogenic differentiation media. In Strategy III, MSCs were cultured with osteogenic induction in monolayer for 12 days, followed by fabricating and culturing spheroids for 2 days with osteogenic induction media. After bioprinting of osteogenic spheroids in a triangular arrangement, the excess amount of alginate microparticles was gently removed as described before. For all strategies, the total differentiation period in the monolayer culture and in the form of spheroids was maintained 14 days in total. Triangle bone constructs were then cultured for another 14 days in osteogenic differentiation media for a total of 28-day culture for all strategies.

### Biological characterization of bioprinted bone tissues.

The bioprinted constructs were visualized by florescent imaging of GFP^+^ MSCs using the Zeiss Axiozoom confocal microscope. H&E and immunohistochemistry staining were carried out to visualize the morphology as described in sections “Histological analysis of spheroids” and “Immunohistochemistry of the bioprinted cartilage tissues,” respectively. For the immunohistochemistry staining, anti-Sp7/OSTERIX primary antibody (1:500 in 2.5% NGS) and goat anti-rabbit Alexa Fluor 568 secondary antibody (1:200 in 2.5% NGS) were used. Samples were imaged using the Zeiss LSM 880 Airyscan Confocal microscope (Zeiss, Oberkochen, Germany). RT-qPCR of bioprinted tissues were conducted for *OSTERIX, COL-1, BSP*, and *BMP-4* genes as described in section “Gene expression of osteogenic spheroids using real time polymerase chain reaction (RT-qPCR).”

### Statistical analysis.

All values were presented as mean ± standard deviation. Multiple comparisons were analyzed by using one-way analysis of variance by post hoc Tukey’s multiple-comparison test was used to determine the individual differences among the groups. Differences were considered significant at *p < 0.05, **p < 0.01, ***p < 0.001, and ****p < 0.0001. All statistical analysis was performed by Statistical Product and Service Solutions software (IBM).

## Data availability

All data needed to evaluate the conclusions in the paper are present in the paper and/or the Supplementary Information. Additional data related to this paper may be requested from the authors.

## Supplementary Material

Supplementary Information

Supplementary Movie 1

Supplementary Movie 2

Supplementary Movie 3

Supplementary Movie 4

Description of Additional Supplementary Files

## Figures and Tables

**Table 1:** Primers of the measured mRNA for real time polymerase chain reaction.

Gene	Forward primer	Reverse primer
*OSTERIX*	CCT CTG CGG GAC TCA ACA AC	AGC CCA TTA GTG CTT GTA AAG G
*COL-1*	ATG ACT ATG AGT ATG GGG AAG CA	TGG GTC CCT CTG TTA CAC TTT
*BMP4*	TAG CAA GAG TGC CGT CAT TCC	GCG CTC AGG ATA CTC AAG ACC
*OCN*	TCA CAC TCC TCG CCC TAT TG	TCG CTG CCC TCC TGC TTG
*BSP*	AAC GAA GAA AGC GAA GCA GAA	TCT GCC TCT GTG CTG TTG GT
*GAPDH*	ATG GGG AAG GTG AAG GTC G	GGG GTC ATT GAT GGC AAC AAT A

## References

[1] Dababneh AB & Ozbolat IT Bioprinting technology: a current state-ofthe-art review. J. Manuf. Sci. Eng 136, 061016 (2014).

[2] Sun W The bioprinting roadmap. Biofabrication 12, 022002 (2020). 32031083 10.1088/1758-5090/ab5158

[3] Leberfinger AN Bioprinting functional tissues. Acta Biomater 95, 32–49 (2019). 30639351 10.1016/j.actbio.2019.01.009 PMC6625952

[4] Ozbolat IT 3D Bioprinting: Fundamentals, Principles and Applications (Academic Press, London, 2016).

[5] Bhattacharjee T Writing in the granular gel medium. Sci. Adv 1, e1500655 (2015). 26601274 10.1126/sciadv.1500655 PMC4643780

[6] Hinton TJ Three-dimensional printing of complex biological structures by freeform reversible embedding of suspended hydrogels. Sci. Adv 1, e1500758 (2015). 26601312 10.1126/sciadv.1500758 PMC4646826

[7] Mattsson J Soft colloids make strong glasses. Nature 462, 83–86 (2009). 19890327 10.1038/nature08457

[8] Saunders BR & Vincent B Microgel particles as model colloids: theory, properties and applications. Adv. Colloid Interface Sci 80, 1–25 (1999).

[9] Dimitriou CJ, Ewoldt RH & McKinley GH Describing and prescribing the constitutive response of yield stress fluids using large amplitude oscillatory shear stress (LAOStress). J. Rheol 57, 27–70 (2013).

[10] Jeon O Individual cell-only bioink and photocurable supporting medium for 3D printing and generation of engineered tissues with complex geometries. Mater. Horiz 6, 1625–1631 (2019). 32864142 10.1039/c9mh00375d PMC7453938

[11] Highley CB, Song KH, Daly AC & Burdick JA Jammed microgel inks for 3D Printing applications. Adv. Sci 6, 1801076 (2019). 10.1002/advs.201801076 PMC6325587 30643716

[12] McCormack A, Highley CB, Leslie NR & Melchels FPW 3D printing in suspension baths: keeping the promises of bioprinting afloat. Trends Biotechnol 38, 584–593 (2020). 31955894 10.1016/j.tibtech.2019.12.020

[13] O’Bryan CS, Bhattacharjee T, Marshall SL, Sawyer WG & Angelini TE Commercially available microgels for 3D bioprinting. Bioprinting 11, e00037 (2018).

[14] O’Bryan CS Self-assembled micro-organogels for 3D printing silicone structures. Sci. Adv 3, e1602800 (2017). 28508071 10.1126/sciadv.1602800 PMC5425239

[15] Ozbolat V, Dey M, Ayan B & Ozbolat IT Extrusion-based printing of sacrificial Carbopol ink for fabrication of microfluidic devices. Biofabrication 11, 034101 (2019). 30884470 10.1088/1758-5090/ab10ae

[16] Jin Y, Compaan A, Bhattacharjee T & Huang Y Granular gel support-enabled extrusion of three-dimensional alginate and cellular structures. Biofabrication 8, 025016 (2016). 27257095 10.1088/1758-5090/8/2/025016

[17] Hinton TJ, Hudson A, Pusch K, Lee A & Feinberg AW 3D printing PDMS elastomer in a hydrophilic support bath via freeform reversible embedding. ACS Biomater. Sci. Eng 2, 1781–1786 (2016). 27747289 10.1021/acsbiomaterials.6b00170 PMC5059754

[18] Pairam E, Le H & Fernández-Nieves A Stability of toroidal droplets inside yield stress materials. Phys. Rev. E 90, 021002 (2014). 10.1103/PhysRevE.90.021002 25215681

[19] Bhattacharjee T Liquid-like solids support cells in 3D. ACS Biomater. Sci. Eng 2, 1787–1795 (2016). 10.1021/acsbiomaterials.6b00218 33440476

[20] Ayan B Aspiration-assisted bioprinting for precise positioning of biologics. Sci. Adv 6, eaaw5111 (2020). 32181332 10.1126/sciadv.aaw5111 PMC7060055

[21] Ayan B, Wu Y, Karuppagounder V, Kamal F & Ozbolat IT Aspiration-assisted bioprinting of the osteochondral interface. Sci. Rep 10, 13148 (2020). 32753630 10.1038/s41598-020-69960-6 PMC7403300

[22] Yu Y Three-dimensional bioprinting using self-assembling scalable scaffold-free “tissue strands” as a new bioink. Sci. Rep 6, 28714 (2016). 27346373 10.1038/srep28714 PMC4921918

[23] Ozbolat IT Scaffold-based or scaffold-free bioprinting: competing or complementing approaches? J. Nanotechnol. Eng. Med 6, 024701 (2015).

[24] Jakab K Tissue engineering by self-assembly of cells printed into topologically defined structures. Tissue Eng. A 14, 413–421 (2008). 10.1089/tea.2007.0173 18333793

[25] Mironov V Organ printing: tissue spheroids as building blocks. Biomaterials 30, 2164–2174 (2009). 19176247 10.1016/j.biomaterials.2008.12.084 PMC3773699

[26] Norotte C, Marga FS, Niklason LE & Forgacs G Scaffold-free vascular tissue engineering using bioprinting. Biomaterials 30, 5910–5917 (2009). 19664819 10.1016/j.biomaterials.2009.06.034 PMC2748110

[27] Mironov V, Boland T, Trusk T, Forgacs G & Markwald RR Organ printing: computer-aided jet-based 3D tissue engineering. Trends Biotechnol 21, 157–161 (2003). 12679063 10.1016/S0167-7799(03)00033-7

[28] Moldovan NI, Hibino N & Nakayama K Principles of the Kenzan method for robotic cell spheroid-based three-dimensional bioprinting. Tissue Eng. Part B Rev 23, 237–244 (2017). 27917703 10.1089/ten.TEB.2016.0322

[29] Gutzweiler L Large scale production and controlled deposition of single HUVEC spheroids for bioprinting applications. Biofabrication 9, 025027 (2017). 28488594 10.1088/1758-5090/aa7218

[30] Style RW, Hyland C, Boltyanskiy R, Wettlaufer JS & Dufresne ER Surface tension and contact with soft elastic solids. Nat. Commun 4, 2728 (2013). 24201430 10.1038/ncomms3728

[31] Becker LE, McKinley GH, Rasmussen HK & Hassager O The unsteady motion of a sphere in a viscoelastic fluid. J. Rheol 38, 377–403 (1994).

[32] Sussman M & Smereka P Axisymmetric free boundary problems. J. Fluid Mech 341, 269–294 (1997).

[33] McKinley GH Steady and transient motion of spherical particles in viscoelastic liquids Transp. Process. Bubbles Drops Part 338–375 (Taylor and Francis, London, 2001).

[34] Gabbanelli S, Drazer G & Koplik J Lattice Boltzmann method for non-Newtonian (power-law) fluids. Phys. Rev. E 72, 046312 (2005). 10.1103/PhysRevE.72.046312 16383538

[35] Chakrabarti A & Chaudhury MK Direct measurement of the surface tension of a soft elastic hydrogel: exploration of elastocapillary instability in adhesion. Langmuir 29, 6926–6935 (2013). 23659361 10.1021/la401115j

[36] Tanner RI Engineering Rheology. CEA, Chemical Engineering in Australia (Oxford University Press, New York, 2000).

[37] Yu Y, Zhang Y, Martin JA & Ozbolat IT Evaluation of cell viability and functionality in vessel-like bioprintable cell-laden tubular channels. J. Biomech. Eng 135, 91011 (2013). 23719889 10.1115/1.4024575 PMC3708706

[38] Heo DN 3D bioprinting of carbohydrazide-modified gelatin into microparticle-suspended oxidized alginate for the fabrication of complex-shaped tissue constructs. ACS Appl. Mater. Interfaces 10.1021/acsami.0c05096 (2020). 32274920

[39] O’Bryan CS, Kabb CP, Sumerlin BS & Angelini TE Jammed polyelectrolyte microgels for 3D cell culture applications: rheological behavior with added salts. ACS Appl. Bio Mater 10.1021/acsabm.8b00784 (2019). 35026924

[40] Song KH, Highley CB, Rouff A & Burdick JA Complex 3D-printed microchannels within cell-degradable hydrogels. Adv. Funct. Mater 28, 1801331 (2018).

[41] Daly AC, Davidson MD & Burdick JA 3D bioprinting of high cell-density heterogeneous tissue models through spheroid fusion within self-healing hydrogels. bioRxiv 10.1101/2020.05.21.103127 (2020). PMC7854667 33531489

[42] Shih Y-RV, Tseng K-F, Lai H-Y, Lin C-H & Lee OK Matrix stiffness regulation of integrin-mediated mechanotransduction during osteogenic differentiation of human mesenchymal stem cells. J. Bone Miner. Res 26, 730–738 (2011). 20939067 10.1002/jbmr.278

[43] Duan B, Yin Z, Hockaday Kang L, Magin RL & Butcher JT Active tissue stiffness modulation controls valve interstitial cell phenotype and osteogenic potential in 3D culture. Acta Biomater 36, 42–54 (2016). 26947381 10.1016/j.actbio.2016.03.007 PMC4883663

[44] Lee WJ Heat shock protein 90 inhibitor decreases collagen synthesis of keloid fibroblasts and attenuates the extracellular matrix on the keloid spheroid model. Plast. Reconstr. Surg 136, 328e–337e (2015). 26313837 10.1097/PRS.0000000000001538 PMC4556118

[45] Wong MY A high-throughput assay for collagen secretion suggests an unanticipated role for Hsp90 in collagen production. Biochemistry 57, 2814–2827 (2018). 29676157 10.1021/acs.biochem.8b00378 PMC6231715

[46] Miri AK Multiscale bioprinting of vascularized models. Biomaterials 198, 204–216 (2019). 30244825 10.1016/j.biomaterials.2018.08.006 PMC6360139

[47] Wu Y Porous tissue strands: avascular building blocks for scalable tissue fabrication. Biofabrication 11, 015009 (2018). 30468153 10.1088/1758-5090/aaec22

[48] Hospodiuk M Sprouting angiogenesis in engineered pseudo islets. Biofabrication 10, 035003 (2018). 29451122 10.1088/1758-5090/aab002

