# Supplementary material for: Aspiration-assisted freeform bioprinting of prefabricated tissue spheroids in a yield-stress gel
Source: Commun Phys. Author manuscript; Available in PMC 2020 Nov 27. (PMC7695349; doi:10.1038/s42005-020-00449-4)
Supplement: Supplementary Information [file NIHMS1645583-supplement-Supplementary_Information.pdf]

Supplementary Information for "Aspiration-assisted Freeform Bioprinting of Pre-fabricated Tissue Spheroids in a Yield-stress Gel"

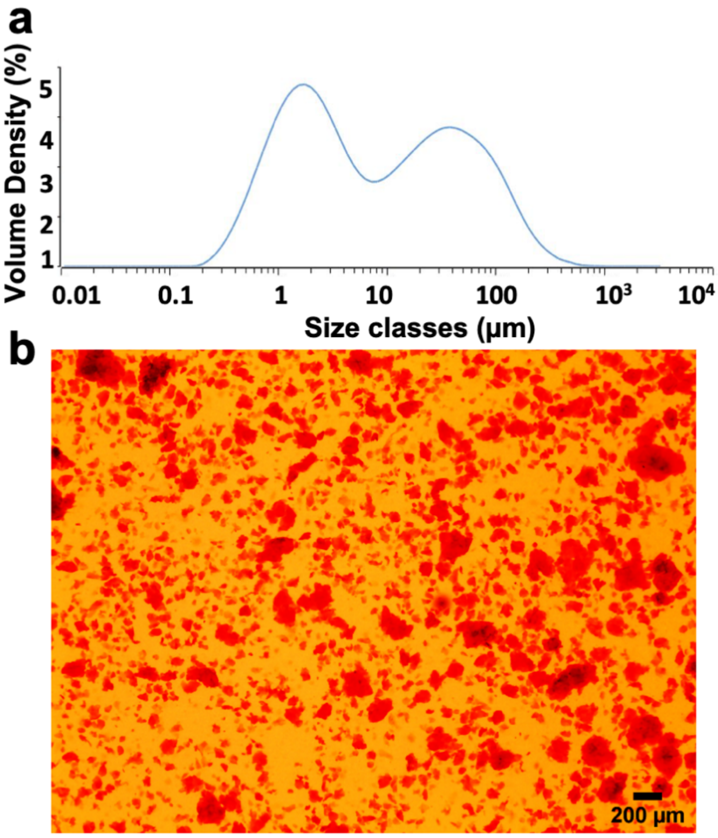

Supplementary Fig. 1. 0.5% alginate microparticles. (a) The mean size of 90% of the particles was determined to be <89.5 µm, which was also corroborated from (b) optical micrograph, showing Safranin-O staining of microparticles.

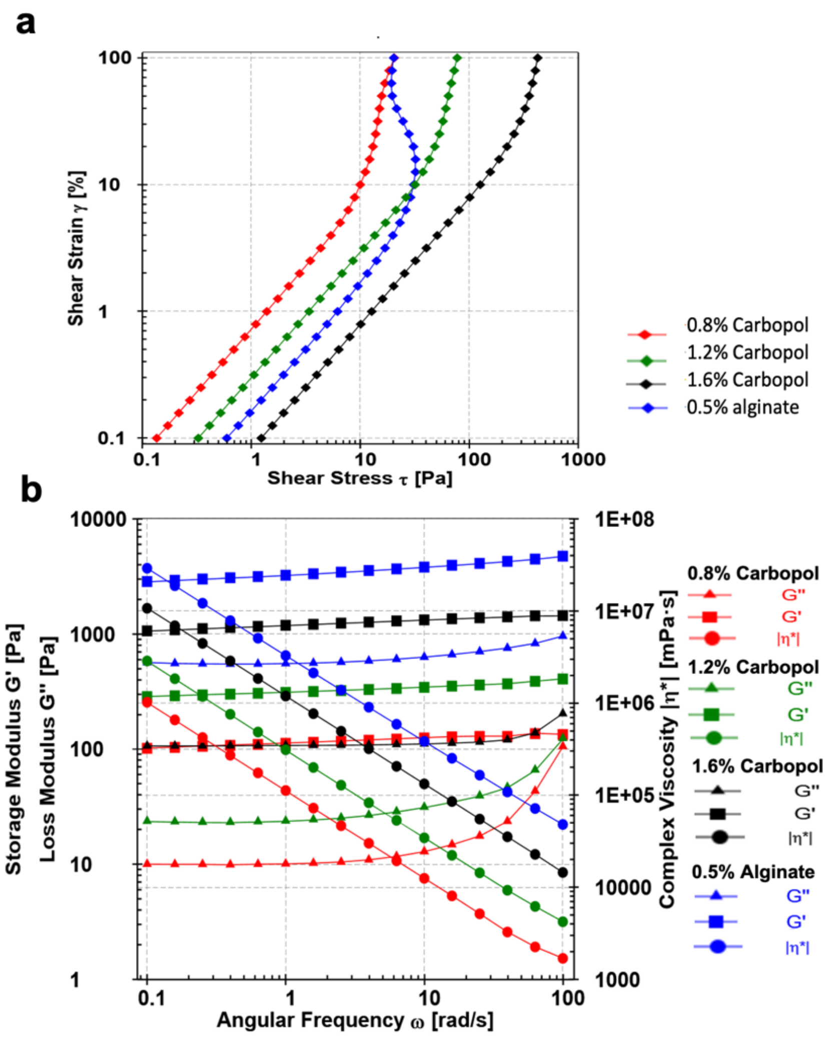

Supplementary Fig. 2. Rheological properties of yield-stress gels at different concentrations. (a) Shear Strain- Shear stress (b) Frequency sweep of yield-stress gels at different concentrations.

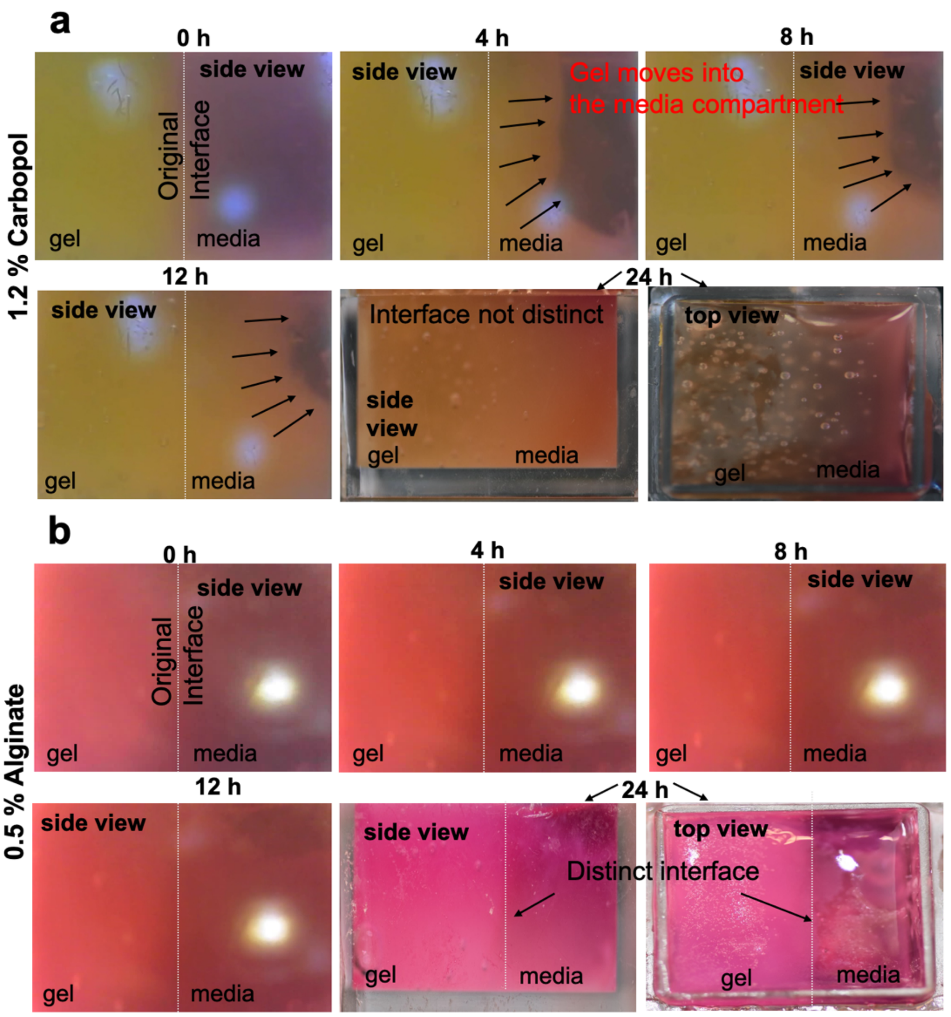

Supplementary Fig. 3. The gradual change of the interface between the media and yield-stress gels. (a) Carbopol gradually flowed into the media. After 24 h, the interface became indistinguishable. (b) Conversely, the alginate microparticles showed a discrete interface with no noticeable degradation after 24 h of device preparation.

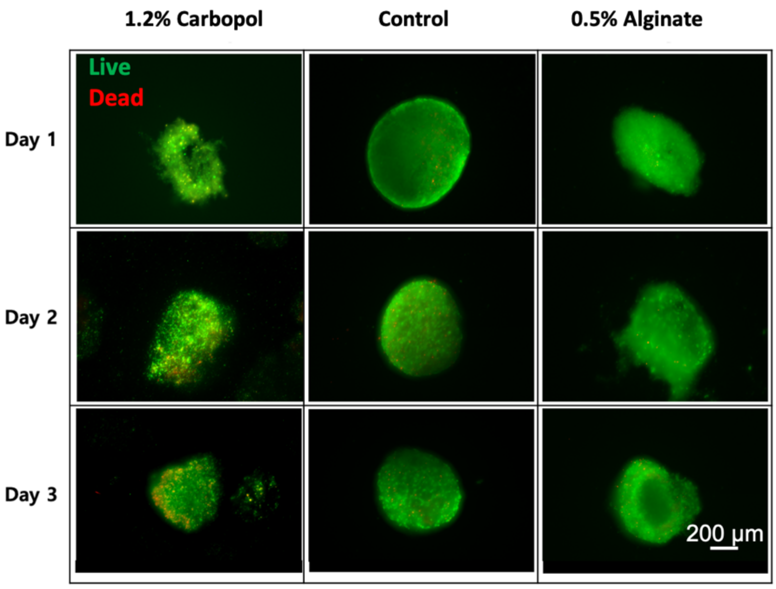

Supplementary Fig. 4. Representative fluorescent images showing the cell viability of mesenchymal stem cell spheroids cultured in the yield-stress gels by LIVE/DEAD assay. Carbopol showed a significant number of dead cells (in red) only 24 h after culture, and the cell viability declined until Day 3, quantified in Fig. 1F. No significant changes in cell viability were noted for the spheroids cultured in 0.5% alginate microparticles.

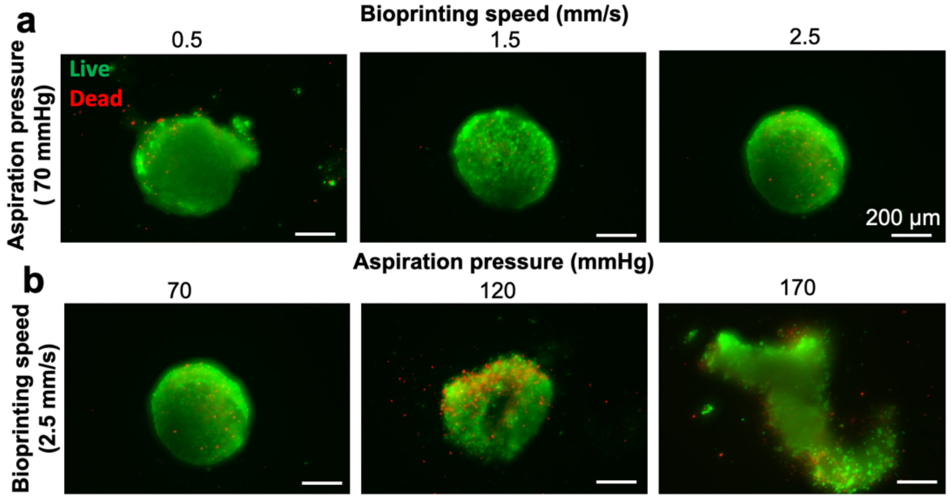

Supplementary Fig. 5. Representative fluorescent images showing the cell viability by LIVE/DEAD assay and circularity of mesenchymal stem cell spheroids with change in the aspiration pressure and bioprinting speed. (a) No significant changes in cell viability and circularity were observed with increase in bioprinting speed under a given aspiration pressure; however, (b) more cell death and deformation was observed with increase in the aspiration pressure.

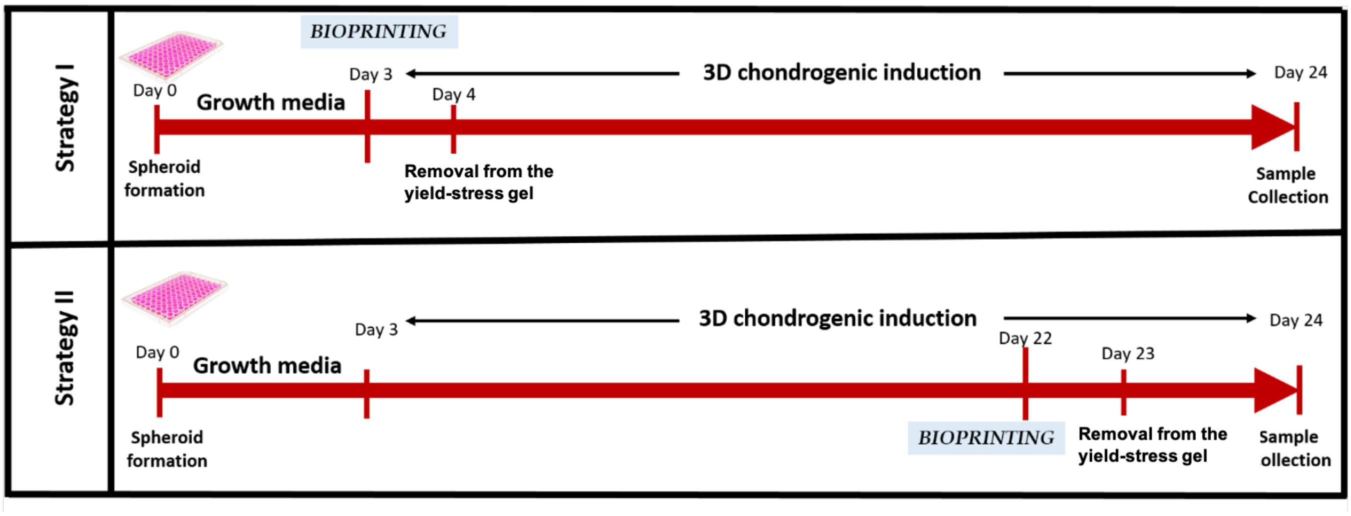

Supplementary Fig. 6. Culture strategies for circular cartilage tissues, including Strategy I and Strategy II

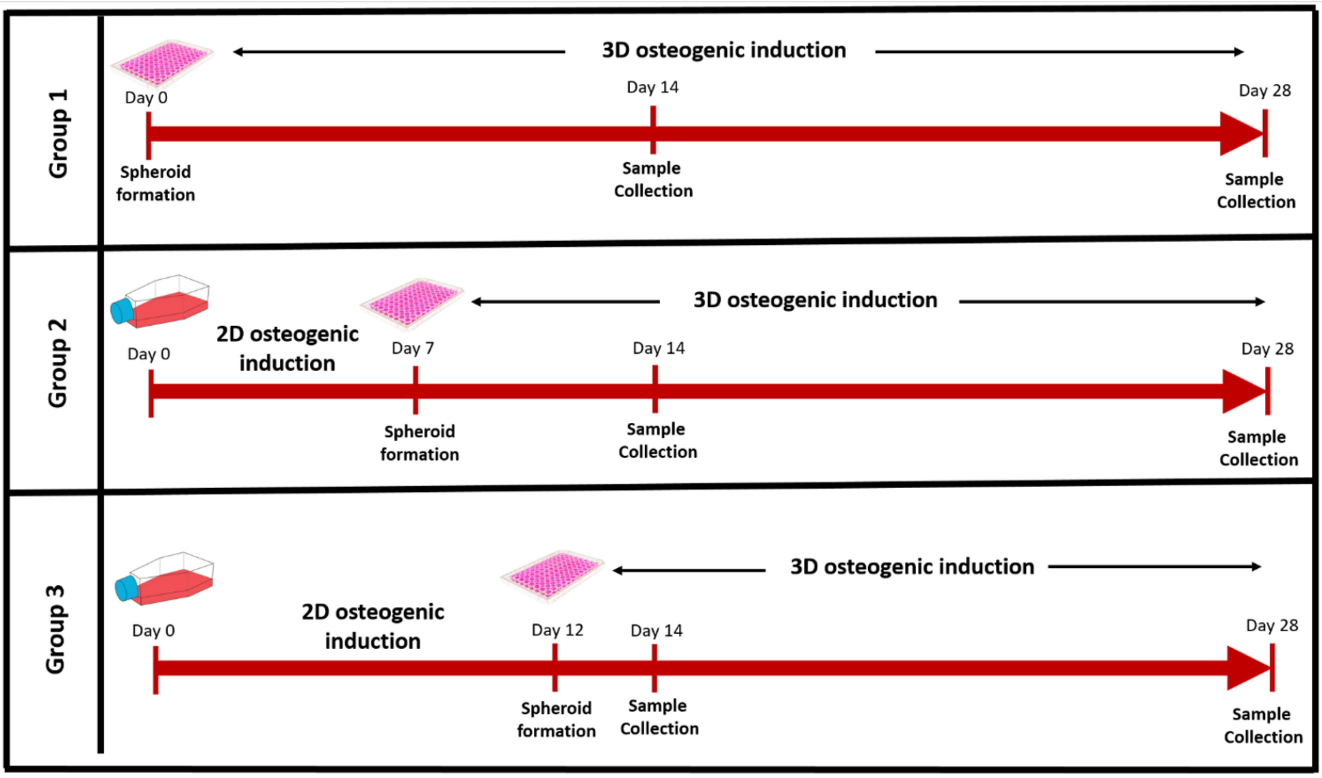

Supplementary Fig. 7. Timeline showing preparation and sample collection of osteogenically-inducted MSC spheroids for Groups 1, 2 and 3.

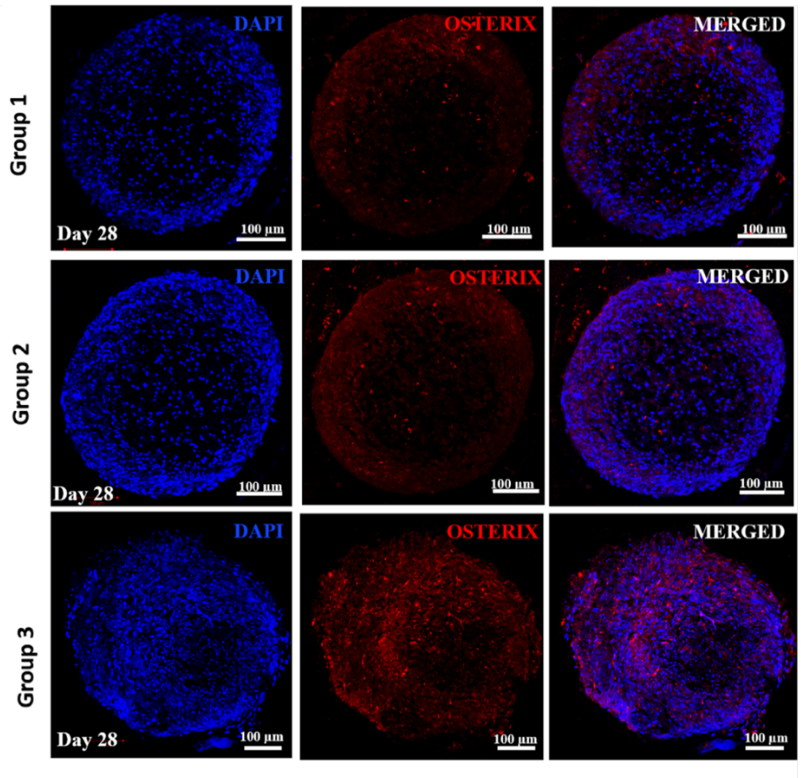

Supplementary Fig. 8. Confocal images of histological sections of different groups of osteogenic spheroids for DAPI, OSTERIX, and DAPI+OSTERIX at Day 28.

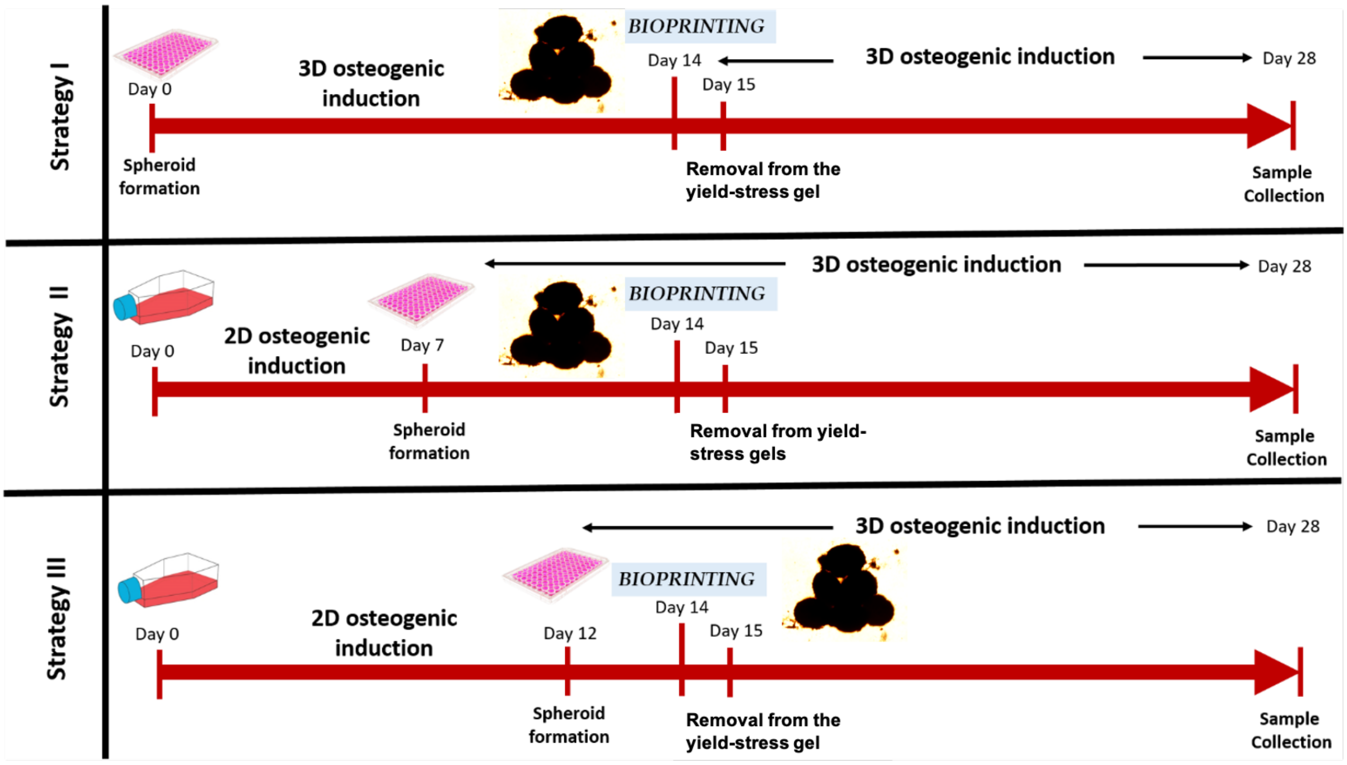

Supplementary Fig. 9. Culture strategies for bioprinted bone tissues including Strategy I, Strategy II, and Strategy III.

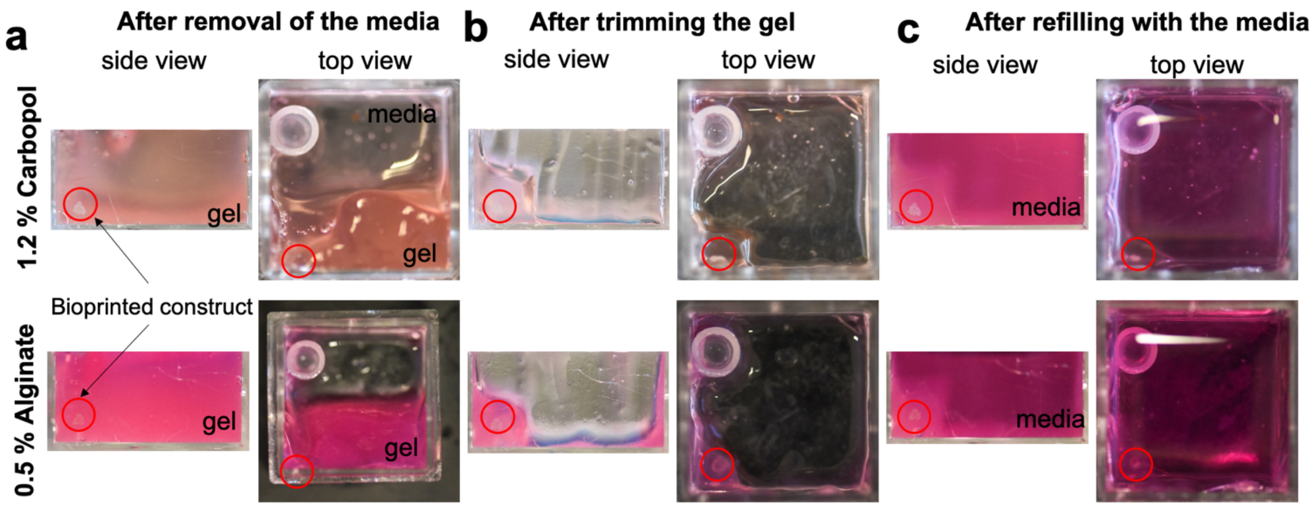

Supplementary Fig. 10. The process of culturing bioprinted constructs in yield-stress gels (1.2 % Carbopol and 0.5 % alginate). After bioprinting, (a) media was first removed from the media compartment, and (b) the gel was carefully trimmed from the sides. Finally, (c) fresh media was added and the constructs were incubated at 37 °C in a humified atmosphere with 5% CO_2.

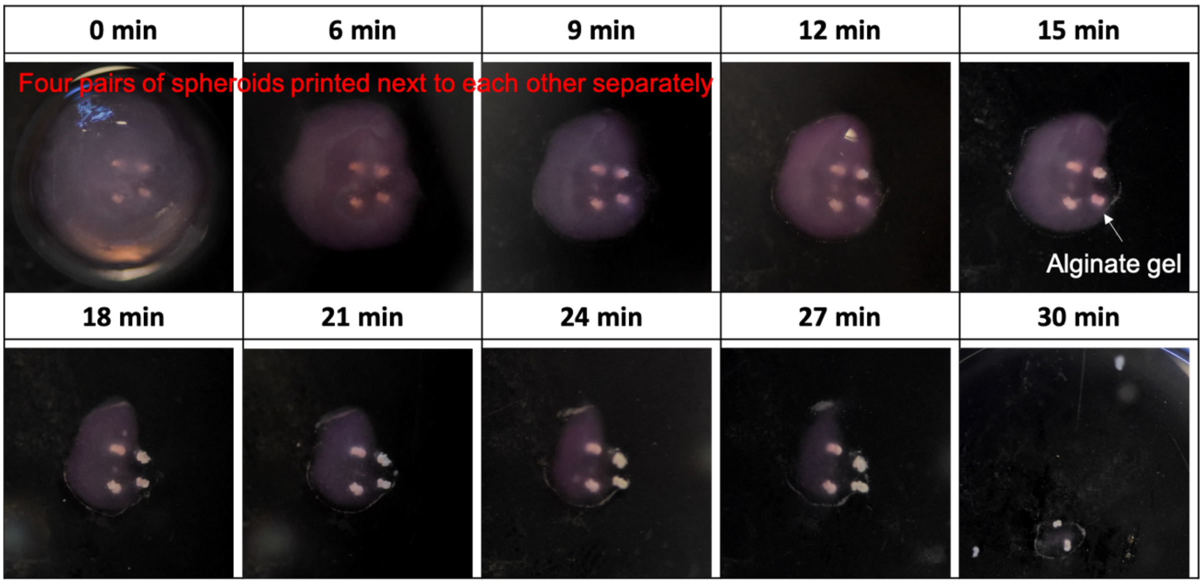

Supplementary Fig. 11. Time-lapse micrographs of the removal of 0.5% alginate with the addition of sodium citrate onto the bioprinted constructs (four pairs of fused spheroids bioprinted next to each other)
