# Supplementary material for: Aspiration-assisted freeform bioprinting of prefabricated tissue spheroids in a yield-stress gel
Source: Commun Phys. Author manuscript; Available in PMC 2020 Nov 27. (PMC7695349; doi:10.1038/s42005-020-00449-4)
Supplement: Description of Additional Supplementary Files [file NIHMS1645583-supplement-Description_of_Additional_Supplementary_Files.pdf]

Description of Additional Supplementary Files

File Name: Supplementary Movie 1
Description: Bioprinting of MSC spheroid pairs in alginate microparticles (40X Speed)

File Name: Supplementary Movie 2
Description: Bioprinting of spheroids in yield-stress Carbopol gel (2X Speed)

File Name: Supplementary Movie 3
Description: A spheroid got stacked at the media-Carbopol interface during bioprinting process

File Name: Supplementary Movie 4
Description: Bouncing of a spheroid at the pipette tip while transitioning in Carbopol
